# Ependymal cells-CSF flow regulates stress-induced depression

**DOI:** 10.1038/s41380-021-01202-1

**Published:** 2021-07-07

**Authors:** Ji-Seon Seo, Ioannis Mantas, Per Svenningsson, Paul Greengard

**Affiliations:** 1 Laboratory of Molecular and Cellular Neuroscience, The Rockefeller University, New York, NY USA; 2 Department of Clinical Neuroscience, Karolinska Institutet, Stockholm, Sweden

**Keywords:** Neuroscience, Molecular biology

## Abstract

Major depressive disorder (MDD) is a severe, common mood disorder. While reduced cerebrospinal fluid (CSF) flow adversely affects brain metabolism and fluid balance in the aging population and during development, only indirect evidence links aberrant CSF circulation with many diseases including neurological, neurodegenerative, and psychiatric disorders, such as anxiety and depression. Here we show a very high concentration of p11 as a key molecular determinant for depression in ependymal cells, which is significantly decreased in patients with MDD, and in two mouse models of depression induced by chronic stress, such as restraint and social isolation. The loss of p11 in ependymal cells causes disoriented ependymal planar cell polarity (PCP), reduced CSF flow, and depression-like and anxiety-like behaviors. p11 intrinsically controls PCP core genes, which mediates CSF flow. Viral expression of p11 in ependymal cells specifically rescues the pathophysiological and behavioral deficits caused by loss of p11. Taken together, our results identify a new role and a key molecular determinant for ependymal cell-driven CSF flow in mood disorders and suggest a novel strategy for development of treatments for stress-associated neurological, neurodegenerative, and psychiatric disorders.

## Introduction

Chronic stress strongly contributes to the manifestation of many diseases including neurological, neurodegenerative, and psychiatric disorders, such as anxiety and depression [1–4]. Our previous studies have implicated p11 (S100A10, annexin II light chain, calpactin I light chain) as an important molecule involved in the etiology of depression [5, 6]. p11 is also implicated in the mechanism of action of antidepressants by directly interacting with multiple type of receptors including 5-HTRs [5, 7], ion channels [8], enzymes, and chromatic-remodeling factors [8, 9]. In addition, p11 regulates stress responses associated with depression by controlling glutamatergic synaptic transmission [10, 11] and hypothalamic-pituitary-adrenocortical (HPA) and sympathetic-adrenal-medullary (SAM) axes activation [12].

Ependymal cells are polarized in the epithelial plane and orient their motile cilia, which is determined by the basal body and basal foot orientation, in a common direction to efficiently propel the cerebrospinal fluid (CSF) [13, 14]. Disruption of ependymal planar cell polarity (PCP) results in aberrant CSF circulation, and reciprocally, external hydrodynamic forces act on intracellular PCP [15–17]. The CSF provides nutrients and neuroendocrine substances, removes toxic metabolites, and preserves the chemical environment of the brain [14, 18]. The CSF flow, which is initiated at the lateral ventricle (LV), maintains CSF homeostasis, and guides neuroblast migration and neurogenesis [14, 19, 20]. The CSF dynamic is highly organized with the glymphatic system for the perivascular exchange of CSF and interstitial fluid (ISF) [21, 22]. Aging and neurological, neurodegenerative, and psychiatric disorders in humans have been associated with abnormal CSF flow [22–26]. However, the underlying molecular and cellular mechanisms are unknown. Here we show that ependymal p11 is critically important for PCP, CSF flow, and depression.

## Materials and methods

### Animals

All procedures involving animals were approved by Karolinska Institutet and The Rockefeller University Institutional Animal Care and Use Committee and were in accordance with the National Institutes of Health guidelines. Eight transgenic mouse lines were generated and used for this study: p11-EGFP mice [10, 27], p11 KO mice [10, 27], Dcdc2a-L10a/EGFP bacTRAP mice (mice were generated from GENSAT), Tppp3-Cre mice (mice were generated from GENSAT), Tppp3-tdT mice (Tppp3-Cre line crossing with tdTomato line), p11 cKO mice (Tppp3-Cre line crossing with p11^f/f^ line [28]), p11 cKO-tdT mice (Tppp3-Cre crossing with tdTomato line and p11 cKO line), and p11 KO (Tppp3) mice (Tppp3-Cre crossing with p11 KO mice). The mouse breeding methods are presented in Supplementary Materials and methods.

### Chronic stress and antidepressant treatments

The restraint, social isolation stress, and antidepressant treatments were performed as previously described [10, 29, 30]. See Methods in Supplementary Materials and methods for details.

### BacTRAP translational profiling

Two or three male Dcdc2a-EGFP/L10a mice and Dcdcd2a-EGFP-L10a crossed with p11 KO mice were used for independent TRAP replicates. The TRAP procedure was performed as described previously [31, 32]. See Methods in Supplementary Materials and methods for details.

### Magnetic resonance imaging (MRI)

Flow and anatomical imaging were performed on a 7.0 Tesla 70/30 Bruker Biospec small animal MRI system (Bruker Biospin, Billerica, MA), yielding transversal maps of regional CSF flow. Flow values were obtained from a region-of-interest analysis using ParaVision 5.1. software (Bruker Biospin). Details are included in Supplementary Materials and methods.

### Ultrastructural analysis

All EM studies were conducted at The Rockefeller University Electron Microscopy Resource Center. Electron microscopy, immuno-electron microscopy (IEM), scanning electron microscopy (SEM), and transmission electron microscopy (TEM) were done as described in Supplementary Materials and methods for details.

### Immunohistochemistry and fluorescence in situ hybridization (FISH)

All immunostaining of human and mouse brain tissue was carried out using the standard method as previously described [10, 28]. Detailed descriptions of antibody preparation, antigen retrieval, image acquisition, and quantification are presented in Supplementary Materials and methods.

### Viruses

The virus production of AAV1-EF1a-DIO-eYFP-WPRE-hGH (AAV_eYFP) and AAV1-EF1a-DIO-p11-WPRE-hGH (AAV_p11) was performed as previously described [10]. See methods in Supplementary Materials and methods for details.

### Behavioral assessments

Behavioral studies including tail suspension test (TST), forced swim test (FST), and novelty suppressed feeding test (NSF) were performed to examine the depression-like and anxiety-like phenotypes, and locomotor activity. Details are included in Supplementary Materials and methods.

### Bioinformatics

RNAseq analysis, gene set enrichment, construction of lateral ventricular ependymal cell-specific functional network, and clustering the depression-associated ependymal cell network were performed. Details are included in Supplementary Materials and methods.

### Statistics

Two-sample comparisons were performed using Student’s *t*-test, while multiple comparisons were made using one-way ANOVA followed by a Newman–Keuls post hoc test or two-way ANOVA by a Bonferroni post hoc test. PRISM software (GraphPad Software) was used to perform statistical analyses. All data are presented as mean ± SEM.

## Results

### Identification of p11 in ependymal cells

**Fig. 1:**
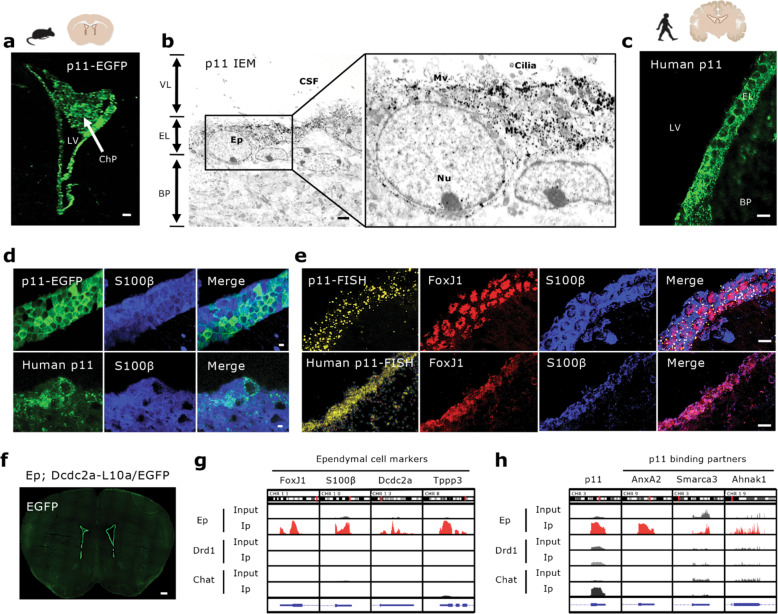
Enrichment of p11 in ependymal cells from mice and humans. **a** Immunofluorescence image illustrating p11-positive cells (EGFP^+^) in the lateral ventricle (LV) and the choroid plexus (ChP) from p11-promoter-driven EGFP (p11-EGFP) mice. Scale bar, 100 μm. **b** Representative image obtained by immuno-electron microscopy using p11 antibody (p11 IEM) illustrating expression of p11 (dots) in ependymal cells in the ventricular–subventricular zone (V–SVZ) of lateral ventricle. VL, ventricle lumen; EL, ependymal cell layer; BP, brain parenchyma; EP, ependymal cells; CSF, cerebrospinal fluid; Nu, nucleus; Mt, mitochondria; Mv, microvilli. Scale bar, 2 μm. **c** Human p11 (green) expression in ependymal cells in the V–SVZ of lateral ventricle. Scale bar, 10 μm. **d** Co-localization of ependymal p11 (green, mouse p11-EGFP^+^, up; and human p11, down) with S100β (blue), as ependymal cell marker in the V–SVZ of lateral ventricle from mice and humans. Scale bars, 10 μm, up; and 5 μm, down. **e** Co-expression of p11 fluorescence in situ hybridization (yellow; mouse p11-FISH, up; and human p11-FISH, down) with FoxJ1 (red) and S100β (blue), ependymal cell markers in the V–SVZ of the lateral ventricle. Scale bar, 30 μm. **f** Immunofluorescence image of EGFP^+^ cells from ependymal cell bacTRAP (Ep; Dcdc2a-L10a/EGFP) mice. Scale bar, 500 μm. **g**, **h** TRAP and RNAseq analysis of cell type-specific translated mRNA expression in ependymal cell bacTRAP (Ep; Dcdc2a-L10a/EGFP) mice, striatal dopamine D1 receptor-expressing neuron-bacTRAP (Drd1; Drd1-L10a/EGFP) mice, and cholinergic neuron-bacTRAP (Chat; ChAT-L10a/EGFP) mice, visualized by Integrative Genomics Viewer (IGV 2.3). Expression of ependymal cell markers (FoxJ1, S100β, Dcdc2a, and Tppp3, **g**), p11 and p11 binding partners (AnxA2, Smarca3 and Ahnak1, **h**) in those cells from the bacTRAP mouse lines.

First, we examined the expression of p11 in ependymal cells using p11 promoter-driven p11-EGFP mice. GFP immunofluorescence was highly concentrated in ependymal cells of the lateral (LV), third (3V), and fourth (4V) ventricles, cerebral aqueduct (Aq), and choroid plexus (ChP) (Fig. 1a and Supplementary Fig. S1a, b). Immuno-electron microscopy (IEM) further confirmed that p11 was selectively enriched in the ependymal cell layer (EL), but not in the ventricular lumen (VL) and brain parenchyma (BP) (Fig. 1b). These results were consistent with p11 expression in human ependymal cells (Fig. 1c). p11 in ependymal cells co-localized with S100β (blue), a marker of ependymal cells (Fig. 1d and Supplementary Fig. S1b). Fluorescence in situ hybridization (FISH) revealed that p11 mRNA (yellow) was also enriched in ependymal cells, which co-localized with FoxJ1 (red) and S100β (blue), as markers of ependymal cells from mice and humans (Fig. 1e and Supplementary Fig. S2a–f).

To determine the translational profile of ependymal cells, we generated a bacTRAP transgenic mouse line (Ep; Dcdc2a-L10a/EGFP) that selectively expressed the ribosomal L10-EGFP subunit in ependymal cells also showing endogenous p11 expression (Fig. 1f and Supplementary Fig. S3a, b). TRAP profiling followed by RNA-sequencing revealed that ependymal cell marker genes including FoxJ1, S100β, Dcdc2a, Tppp3, and p11, along with p11 binding partners, such as AnxA2, Smarca3, and Ahnak1, were more abundant in ependymal cells compared to other cell types enriched in p11, such as striatal dopamine D1 Receptor-expressing neurons (Drd1; Drd1-L10a/EGFP) [31] and cholinergic interneurons (Chat; ChAT-L10a/EGFP) [31] (Fig. 1g, h). These data indicate that p11 is highly enriched in mouse and human ependymal cells.

### Chronic stress induces the loss of p11 in ependymal cells, depression-like behaviors and disruption of CSF flow

**Fig. 2:**
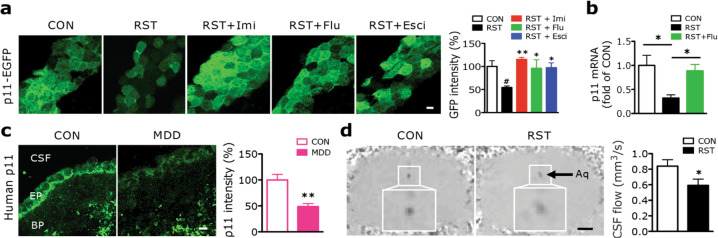
Chronic stress decreases p11 expression in ependymal cells and reduces CSF flow. **a** Immunofluorescence images illustrating p11-EGFP-positive ependymal cells in the V–SVZ of lateral ventricle from control (CON) and chronic restraint stressed (RST) mice with or without antidepressants. Imi; imipramine, Flu; fluoxetine, Esci; escitalopram. Scale bar, 5 μm. Quantification of p11 expression (EGFP^+^) in ependymal cells from those mice (*n* = 8 for CON, *n* = 5 for each group of RST, RST + Imi, RST + Flu, RST + Esci). **b** Expression of p11 mRNA in LV ependymal cells TRAP and qPCR measurements from CON, RST and RST + Flu ependymal cell bacTRAP mice (*n* = 10 for CON, *n* = 12 for each group of RST and RST + Flu). **c** Ependymal p11 expression in the V–SVZ of lateral ventricle and quantification from patients with major depressive disorder (MDD) and unaffected control (CON) from postmortem brain sections (*n* = 15 for each group). CSF, cerebrospinal fluid; EP, ependymal cells; BP, brain parenchyma. Scale bar, 10 μm. See Supplementary Table S1 for the detailed sample information. **d** Magnetic resonance imaging (MRI) images represent CSF flow in the cerebral aqueduct (Aq) from control and stressed mice. Scale bar, 850 μm. Quantification of CSF flow from the control and stressed mice (*n* = 7 for each group). ^#^*P* < 0.05, compared to CON; *P < 0.05 and **P < 0.01, compared to RST, ANOVA test. *P < 0.05 and **P < 0.01, Student’s *t*-test. Data are mean ± s.e.m.

Next, we investigated whether p11 expression in ependymal cells is altered by stress. Mice exposed to chronic restraint stress (RST, 2 h/day, 14 days) [29], which have shown depression-like behaviors [10], exhibited significantly reduced p11-EGFP expression in ependymal cells compared to the non-stressed control group (CON), and this downregulation was reversed by a 2-week treatment with either of three different antidepressants: imipramine (TCA), fluoxetine (SSRI), or escitalopram (SSRI) (Fig. 2a). These data were confirmed by TRAP profiling followed by quantitative PCR (qPCR), and immunofluorescence staining with p11 antibody (Fig. 2b and Supplementary Fig. S4), indicating that chronic RST induces the loss of p11 in ependymal cells, which is reversed by antidepressants.

We further confirmed that chronic stress alters p11 expression in ependymal cells using another stress stimulus. Mice exposed to chronic social isolation stress (IS, 8 weeks) [30] exhibited significantly decreased p11-EGFP expression in ependymal cells, and significantly increased immobility in the tail suspension test (TST) and forced swim test (FST), two measurements of helplessness and hopelessness (Supplementary Fig. S5a–c). Taken together, these data support the hypothesis that the loss of p11 in ependymal cells contributes to the manifestation of depression-like behaviors in response to stress.

To determine the clinical relevance of these findings, we investigated p11 expression in ependymal cells in postmortem brain sections from major depressive disorder (MDD) patients versus unaffected control subjects (CON). Importantly, MDD patients showed significantly reduced p11 expression in ependymal cells, but not in brain parenchyma (BP) (Fig. 2c and Supplementary Table S1), indicating that the loss of p11 in ependymal cells is associated with depression in humans.

Given the abnormalities of CSF flow in depressed patients [25], we sought to determine whether the stress-induced loss of ependymal p11 is responsible for CSF flow. Using MRI, we measured the CSF flow in the cerebral aqueduct (Aq) of live mice. Compared to the non-stressed control (CON) group, mice exposed to chronic restraint stress (RST) exhibited significantly decreased CSF flow (Fig. 2d).

### The expression of p11 in ependymal cells determines planal cell polarity and CSF flow

**Fig. 3:**
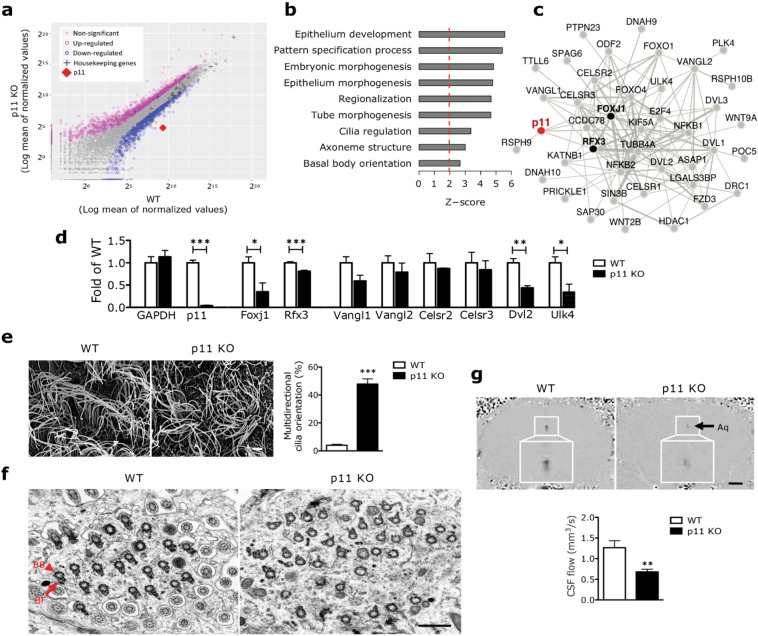
p11 regulates ependymal cell planar cell polarity and CSF flow. **a** Scatter plot displaying ependymal cell translational profiling in the lateral ventricle (LV) from WT (Dcdcd2a-L10a/EGFP) and p11 KO (p11 KO x Dcdc2a-L10a/EGFP) bacTRAP mice (*n* = 12, WT; *n* = 8, p11 KO). See Supplementary Table S2 for numerical data. **b** Rank-based gene set enrichment of selected planar cell polarity (PCP)-related processes on p11-regulated LV ependymal cell gene differential expression, presented as *z*-score (positive *z*-scores indicate upregulation of processes in WT compared to p11 KO). See Supplementary Fig. S7 and Supplementary Tables S3 and S4 for numerical data. **c** The LV ependymal PCP core genes functional interaction network with p11; only functional interactions with larger than three fold over random are shown. See Supplementary Table S5 for additional data compendium used to build the network. **d** Expression of PCP gene mRNA in the ependymal cell-specific TRAP profiling from WT and p11 KO bacTRAP mice (*n* = 10, WT; *n* = 16, p11 KO). **e** Scanning electron microscopy (SEM) images illustrating ependymal cell PCP orientation in the V–SVZ of lateral ventricle from WT and p11 KO mice. Quantification of multidirectional cilia orientation in LV ependymal cells from WT and p11 KO mice (*n* = 20 cells from 3 mice in each group). BB, basal body (arrowhead); BF, basal foot (arrow). Scale bar, 2 μm. **f** Transmission electron microscopy (TEM) images illustrating bosal body and basal foot orientation in ependymal cells in the V–SVZ of lateral ventricle from WT and p11 KO mice. **g** MRI images represent CSF flow and quantification of CSF flow from p11 KO mice and WT littermates (*n* = 7 for each group). Scale bar, 850 μm. **P < 0.01, ***P < 0.001, Student’s *t*-test. Data are mean ± s.e.m.

To investigate the molecular mechanisms by which p11 regulates ependymal cell function, we performed TRAP profiling of ependymal cell-specific bacTRAP (Dcdc2a-L10a/EGFP) mice crossed to constitutive p11 KO mice. Loss of p11 significantly changed the translational levels of a large number of genes expressed in ependymal cells with respect to wildtype littermates (2240 mRNAs upregulated and 1803 mRNAs downregulated, Fig. 3a, Supplementary Fig. S6, and Supplementary Table S2). Rank-based gene set enrichment analysis revealed that loss of p11 significantly decreased planar cell polarity (PCP)-related processes (Fig. 3b, Supplementary Fig. S7, and Supplementary Tables S3 and S4). These data indicate that p11 is intrinsically linked to the regulation of PCP in ependymal cells. Moreover, we constructed an ependymal cell functional gene interaction network (See Materials and methods for details), and network analysis revealed that p11 has strong functional interaction with several PCP core genes, such as FoxJ1 and Rfx3, the master genes of cilia and PCP (Fig. 3c and Supplementary Table S5).

PCP genes control cilia orientation, which is required for proper CSF flow [13, 15, 17]. Ependymal cell-specific TRAP profiling revealed that FoxJ1, Rfx3, Dvl2, and Ulk4, as rotational PCP regulators [33, 34], were significantly decreased from p11 KO bacTRAP mice compared to wildtype littermates (Fig. 3d). Scanning electron microscopy (SEM) imaging revealed that loss of p11 induced rotational PCP defects measured by significantly increased multidirectional and decreased unidirectional cilia orientation in ependymal cells from p11 KO and stressed mice (Fig. 3e and Supplementary Figs. S8a, b and S9a–c). Transmission electron microscopy (TEM) imaging further confirmed that loss of p11 induces ependymal PCP disorientation demonstrated by increased multidirectional and multiple basal foot, which dictates cilia orientation and CSF flow direction, from p11 KO mice compared to wildtype littermates (Fig. 3f). The ependymal PCP disorientation in p11 KO mice is associated with decreased CSF flow (Fig. 3g). Immunofluorescence and immuno-EM (IEM) analyses indicated that, within the ependymal cell layer, p11 is expressed in cilia from mice and humans (Supplementary Fig. S10a–c). The number of cilia in stressed and p11 KO mice, and cilia length, axonemal structure and diameter, as well as ventricle size, were not altered in p11 KO mice compared to wildtype (Supplementary Figs. S8c, d, S9d, S11, and S12). These data show that p11 in ependymal cells may control CSF flow by regulating ependymal PCP, basal foot and cilia orientation.

Given a close correlation with demonstrated between CSF flow and depressive behaviors in response to loss of p11 expression in ependymal cells, we sought to determine a causal relationship between ependymal cells and depression. We created a depression-associated ependymal cell functional module (Supplementary Fig. S13 and Supplementary Tables S6 and S7). Eight clusters revealed that ependymal cells are strongly involved in regulation of depression with Ca^2+^ signaling, ion and protein transport, neuronal fate regulation, epithelial cell proliferation, learning and memory and fluid regulation. These clusters show similar trends in the ependymal p11-mediated signaling pathways (Supplementary Fig. S7). These results indicate that ependymal cells may regulate depression.

### p11 overexpression in ependymal cells ameliorates stress-induced pathophysiological and behavioral deficits

To determine the role of p11 in ependymal cells, we generated conditional knockout mice with p11 deletion in ependymal cells (p11 cKO; Tppp3-Cre x p11^f/f^, Supplementary Fig. S14). Compared to the wildtype littermates, p11 cKO mice exhibited significantly decreased CSF flow and disoriented ependymal PCP demonstrated decreased unidirectional and increased multidirectional cilia orientation, while number of cilia number was not altered (Supplementary Fig. S14b–e). The p11 cKO mice displayed depression-like behaviors measured by increased immobility in TST and FST, and anxiety-like behavior shown as increased latency to feed in novelty suppressed feeding (NSF), while locomotor activity was not altered (Supplementary Fig. S14f–i). These data indicate that selective loss of p11 in ependymal cells contributes to disoriented ependymal PCP and decreased CSF flow, as well as depression-like and anxiety-like behaviors.

**Fig. 4:**
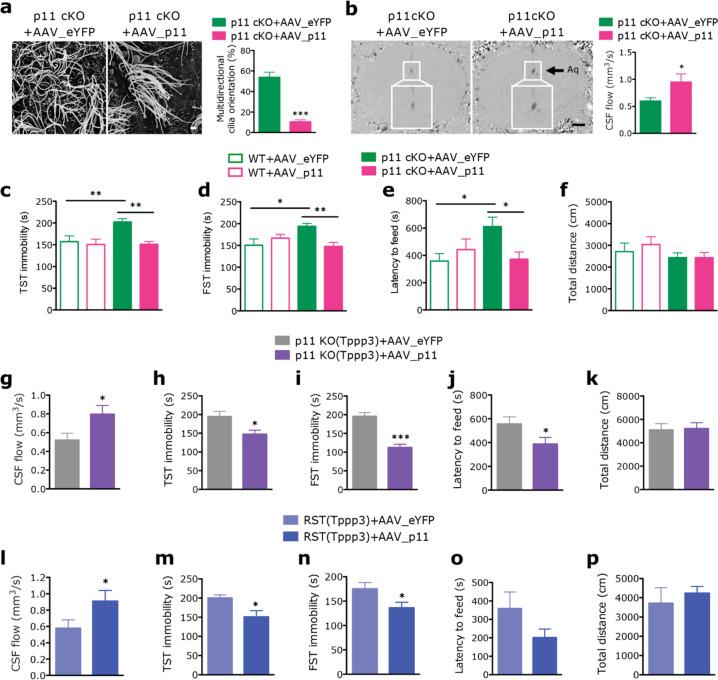
Overexpression of p11 in ependymal cells from depressive mice ameliorates disrupted PCP and CSF flow, as well as depression-like behaviors. **a** SEM images and quantification of multidirectional cilia orientation in ependymal cells in the V–SVZ of lateral ventricle from conditional p11 knockout (p11 cKO; Tppp3-Cre x p11^f/f^) mice with Cre-dependent viral overexpression of AAV1-DIO-p11 (AAV_p11) or AAV1-DIO-eYFP (AAV_eYFP) into intracerebroventricularly (*n* = 11 for each group). Scale bar, 2 μm. **b** MRI images and quantification of CSF flow from p11 cKO with the expression of AAV_p11 or AAV_eYFP in ependymal cells (*n* = 7, p11 cKO+AAV_eYFP; *n* = 8, p11 cKO+AAV_p11). Scale bar, 850 μm. **c**–**f** Depression- and anxiety-like behaviors in WT and p11 cKO mice with viral overexpression of AAV_p11 in ependymal cells, as measured by immobility time in tail suspension test (TST, **c**), forced swim test (FST, **d**), the latency to feed in novelty suppressed feeding test (NSF, **e**), and the total travel distance in locomotor activity test (**f**) (*n* = 10, WT + AAV_eYFP and WT + AAV_p11; *n* = 14, p11 cKO+AAV_eYFP and p11 cKO+AAV_p11). **g** Quantification of CSF flow from constitutive p11 KO crossed to Tppp3-Cre [p11 KO (Tppp3); p11 KO x Tppp3-Cre] mice with viral overexpression of AAV_p11 or AAV_eYFP into intracerebroventricularly. **h**–**f** Depression-like behaviors in p11 KO (Tppp3) mice with viral overexpression of AAV_p11 or AAV_eYFP in ependymal cells, as measured by TST (**h**), FST(**i**), NSF(**j**), and locomotor activity (**k**) (**g**–**k**, *n* = 8, p11 KO (Tppp3)+AAV_eYFP; *n* = 9, p11 KO (Tppp3)+AAV_p11). **l** Quantification of CSF flow from chronic stressed Tppp3-Cre [RST(Tppp3)] mice with viral overexpression of AAV_p11 or AAV_eYFP into intracerebroventricularly. (*n* = 5, RST(Tppp3)+AAV_eYFP; *n* = 8, RST(Tppp3)+AAV_p11). **m**–**p** Depression-like behaviors in WT and p11 KO (Tppp3) mice with viral overexpression of AAV_p11 in ependymal cells, as measured by TST (**m**), FST(**n**), NSF (**o**), and locomotor activity (**p**) (*n* = 8, RST(Tppp3)+AAV_eYFP; *n* = 7, RST(Tppp3)+AAV_p11). *P < 0.05 and ***P < 0.001, Student’s *t*-test. *P < 0.05 and **P < 0.01, ANOVA test. Data are mean ± s.e.m.

The Cre-dependent viral re-expression of p11 (AAV_p11; AAV1-DIO-p11) in ependymal cells specifically from p11 cKO mice reversed the disruption of ependymal PCP, cilia orientation, and CSF flow, but did not change the number of cilia (Fig. 4a, b and Supplementary Fig. S15a–d). The depression-like and anxiety-like behaviors of p11 cKO mice observed in TST, FST, and NSF tests were also rescued by viral overexpression of p11 in ependymal cells, while locomotor activity was not altered (Fig. 4c–f).

Constitutive p11 knockout (p11 KO) mice also exhibited decreased CSF flow, disrupted ependymal PCP, and cilia disorientation, as well as depressive behavior [10] (Fig. 3). Viral re-expression of p11 (AAV_p11) in ependymal cells of p11 KO crossed to Tppp3-Cre [p11 KO(Tppp3)] mice rescued the decreased CSF flow and depression-like behavioral phenotypes, while locomotor activity was not altered (Fig. 4g–k).

Moreover, we examined whether restoring p11 in ependymal cells could reverse stress-induced depression and decreased CSF flow, in parallel with the loss of p11 in ependymal cells (Fig. 2a, b, d and Supplementary Figs. S5a–c and S9a–c). Interestingly, overexpressing AAV_p11 in ependymal cells of stressed Tppp3-Cre [RST(Tppp3)] mice ameliorated stress-induced pathophysiological and behavioral deficits (Fig. 4l–p).

Collectively, these data show that restoration of p11 expression in ependymal cells is sufficient for the reversal of the decreased CSF flow and depressive behaviors induced by chronic stress and genetically manipulated mice with ependymal cells or constitutive deletion of p11, indicating that p11 is a key molecular determinant for ependymal cells in the regulation of CSF flow and depression.

## Discussion

Chronic stress underlies the development of many diseases including neurological, neurodegenerative, and psychiatric diseases, such as anxiety and depression [1, 2, 35–37]. Our previous studies have shown that p11 is a key causal factor for depression, and mediates stress responses and antidepressant actions in mice and humans [8]. p11 is present in various neuronal circuits in distinct neuronal types, such as cholinergic neurons in nucleus accumbens [28], mossy cells and basket cells in dentate gyrus [9], and layer 5 corticostriatal projection neurons [38], involved in emotional control. Downregulated p11 mRNA and protein have been found in the brains of depressed humans and suicide subjects [5, 39]. p11 KO mice exhibit depression-like behaviors, and p11 overexpressing mice show antidepressant-like behaviors [5, 8]. Moreover, chronic stress induces a downregulation of p11 expression in the dopamine D2 receptor (D2R)-expressing neurons in layer 2/3 prelimbic (PrL) and an upregulation of p11 expression in the medial part of the lateral habenula (LHb) resulting in attenuation of glutamatergic synaptic transmission in PrL [10] and hyperexcitability of LHb neurons [11]. p11 in vasopressinergic cells of paraventricular nucleus mediates stress-induced activation of HPA and SAM axes [12].

In the present study, we have identified a novel molecular mechanism by which the control of ependymal cells regulates the depressive state. We have shown here that decreased CSF flow and depressive behavioral phenotypes in chronically stressed animals and MDD patients are closely associated with the reduction of p11 in ependymal cells. p11 intrinsically controls PCP core genes, which mediates ependymal PCP, CSF flow, and depression-like and anxiety-like behaviors. Overexpression of p11 in ependymal cells rescues the pathophysiological and behavioral deficits in depressed mice induced by chronic stress or constitutive or ependymal cell-specific genetic deletion of p11 (Supplementary Fig. S16).

The ependymal cells, which forms a barrier between the brain parenchyma and the CSF, may be reciprocally regulated by CSF and blood vessels through B1 cells, which are neural stem cells in the ventricular–subventricular zone (V–SVZ), and various innervating axons, such as supraependymal serotonergic axons that contribute to migrating neuroblasts and adult neurogenesis in the V–SVZ of lateral ventricle [19, 40]. Cholinergic [41] and dopaminergic [42] axon terminals are also observed in this area. The CSF flow maintains CSF homeostasis by providing nutrients and neuroendocrine substances, removing toxic metabolites, and preserving the chemical environment of the brain, and guides neuroblast migration and adult neurogenesis [13, 20, 43]. In parallel, stress-induced psychiatric disorders are associated with decreased adult neurogenesis and CSF flow [3, 20, 25, 44].

The control of CSF flow may impact the neurological, neurodegenerative, and psychiatric diseases through the integrated action of the ependymal cell milieu, including CSF and blood vessels, glymphatic system, supraependymal serotonergic, cholinergic and dopaminergic axons, and neural circuits [14, 19, 21, 22]. In addition, CSF flow is regulated by respiration [45]. However, to our knowledge, there is currently no strong evidence that p11 is involved in physiological mechanisms that significantly affects respiration or PO_2_ and CO_2_ in blood. Under posthypoxic conditions, pulse oximetry estimation of hemoglobin saturation of PO_2_ and CO_2_ are the same for wildtype and p11 KO mice [46].

Ependymal cell-specific TRAP profiling reveals that p1l controls not only ependymal PCP, but also 5-HT signaling pathway, ion, neurotransmitter, hormone and neuropeptide transport and secretion, chromatin modification, synaptic transmission, immune responses, and learning and memory (Fig. 3c, Supplementary Fig. S7, and Supplementary Table S4), which are closely involved in depression. These data further confirm with the depression-associated ependymal cell functional modules from mice and humans and suggest that ependymal cells have a close correlation with depression (Supplementary Fig. S13 and Supplementary Table S7). It is interesting to note that not only p11, but also several of its known binding partners are expressed in ependymal cells (Fig. 1h). However, given that the expression of these binding partners is not altered in ependymal cells from p11 KO mice (Supplementary Table S2), it is unlikely that they are involved in ependymal p11 actions in depression. To better understand the molecular mechanisms whereby ependymal p11 regulates depression-like states, we plan, in future work, on determining 5-HT levels as well as interactions between p11 and 5-HT receptors in ependymal cells in stressed and depressed mice. We plan also to examine CSF flow and depressive-like behaviors in mice with genetically altered p11 in supraependymal serotonergic axons.

Collectively, our data suggest that p11 is a key molecular determinant for ependymal cells in the regulation of CSF flow and depression. The detailed mechanisms by which ependymal p11 directly or indirectly regulates the association of CSF flow and depression remain to be determined. Elucidation of the specific pathways involved should enable the identification of new targets for the treatment of depression. This and future work on the role of ependymal cells in the control of CSF flow, depression, and anxiety will provide framework for the development of novel strategies to treat neurological, neurodegenerative, and psychiatric diseases.

## Supplementary information

Supplementary Information

Supplementary Table S1

Supplementary Table S2

Supplementary Table S3

Supplementary Table S4

Supplementary Table S5

Supplementary Table S6

Supplementary Table S7
