# Supplementary material for: Ependymal cells-CSF flow regulates stress-induced depression
Source: Mol Psychiatry. 2021 Jul 7;26(12):7308–15. doi: 10.1038/s41380-021-01202-1 (PMC8873010; doi:10.1038/s41380-021-01202-1)
Supplement: Supplementary file 1 — Supplementary Information [file 41380_2021_1202_MOESM1_ESM.pdf]

Supplementary Information for

Ependymal cells-CSF flow Regulates Stress-induced Depression

Ji-Seon Seo[1,2,*], Ioannis Mantas[2], Per Svenningsson[2], Paul Greengard[1]

[1]Laboratory of Molecular and Cellular Neuroscience, The Rockefeller University, New York, NY 10065, USA. [2]Department of Clinical Neuroscience, Karolinska Institutet, 171 77 Stockholm, Sweden. [*]Corresponding author. e-mail: jiseon.seo@ki.se.

This PDF file includes:

Supplementary Materials and Methods
Supplementary Figure Legends
Supplementary Figures S1 to S16
Supplementary References

Other Supplementary information for this manuscript include for following:

Supplementary Tables S1 to S7 (Excel)

SUPPLEMENTARY MATERIALS AND METHODS

Animals

Eight transgenic mouse lines were generated and used for this study: **p11-EGFP** mice[10, 27], **p11 KO** mice[10, 27], **Dcdc2a-L10a/EGFP** bacTRAP mice (Mice were generated from GENSAT, by modifying a BAC clone containing the Dcdcd2a gene to insert an EGFP-L10a fusion protein into the translation start site, and the modified BAC was purified and used for transgenes as described previously[27], **Tppp3-Cre** mice (Mice were generated from GENSAT, by modifying a BAC clone containing the Tppp3 gene to insert Cre recombinase into the translation start site, and the modified BAC was purified and used for transgenes as described previously[27]. Tppp3-Cre-expressing cells are detected in the prefrontal cortex, the cerebellum and the brainstem). **Tppp3-tdT** mice (Tppp3-Cre line crossing with tdTomato line), **p11 cKO** mice (Tppp3-Cre line crossing with $p11^{f/f}$ line[28]), **p11 cKO-tdT** mice (Tppp3-Cre crossing with tdTomato line and p11 cKO line) and **p11 KO (Tppp3)** mice (Tppp3-Cre crossing with p11 KO mice). The C57BL/6J mice and tdTomato reporter mice (Rosa26-CAG-$tdTomato^{loxp/+}$, 007908) were purchased from the Jackson Laboratory. We produced the progeny for each line by in vitro fertilization (IVF) and embryo transfer (ET) techniques (Transgenic Facility, The Rockefeller University, New York, NY, USA). All experiments were approved by Karolinska Institutet and The Rockefeller University Institutional Animal Care and Use Committee and were performed in accordance with the guidelines described in the National Institutes of Health Guide for the Care and Use of Laboratory Animals. Mice were housed in groups of up to five animals on a 12 h dark/light cycle at 22°C and maintained with rodent diet (Picolab) and water available ad libitum. Male mice were used for all experiments.

Chronic stress and antidepressant treatments

Restraint stress treatment was performed as described previously[10, 29]. Briefly, mice were housed 2 per cage and individually placed head-first into well ventilated 50 ml polypropylene conical tubes, which were then plugged with a 4.5-cm-long middle tube, and finally tied with the cap of the 50 ml tube. After each session of restraint stress, the mice were returned to their home environment, in which they were housed in pairs in normal plastic cages with free access to food and water. Isolation stress treatment was performed as described previously[30]. Briefly, mouse was single housed for 8 weeks. From the next day following the last restraint session,

imipramine, fluoxetine and escitalopram were administered daily intraperitoneal injection (i.p.) injection (20 mg/kg/day) for 2 weeks. Imipramine, fluoxetine and escitalopram were purchased from Sigma-Aldrich. Fluoxetine and escitalopram were dissolved in dimethylsulfoxide (DMSO) and then diluted in saline. Imipramine was dissolved in saline. Each drug was finally diluted in 100 µl of 0.9% saline and administered at the dose indicated. Control groups were administered saline.

BacTRAP Translational profiling

Two or three male Dcdc2a-EGFP/L10a mice and Dcdcd2a-EGFP-L10a crossed with p11KO mice were used for independent TRAP replicates. The TRAP procedure was performed as described previously[31, 32]. In brief, tissues that included lateral ventricles were dissected from individual mice. Tissue was immediately homogenized with a motor-driven Teflon glass homogenizer. Polyribosomes were immunoprecipitated by two monoclonal anti-EGFP antibodies (19C8 and 19F7) with coated protein L magnetic beads. RNAs from polyribosomes were extracted and further purified with a Rneasy Plus Micro Kit. RNA quantity and quality were determined with an Agilent 2100 Bioanalyzer. cDNA was synthesized from 5 ng of mRNA from IP and input samples and further amplified using a Oviation RNA-seq Kit. cDNA fragments of 200 bp were end-repaired and ligated with adapters for HiSeq 2000 (Ilumina Inc., San Diego, CA, USA) technology using TruSeq Nano DNA Sample kit (Illumina). Quality of libraries was assessed using a HT DNA High Sensitivity Chip (Agilent) and a 2100 Bioanalyzer. RNA-seq reads were aligned to the UCSC mm10 reference genome using STAR (version 2.3.0e_r291). Aligned reads were quantified by htseq-count module, part of the 'HTSeq' framework (version 0.6.0). Differentially expressed genes were identified by performing a negative binomial test using DESeq2 (R-package version 1.4.5) with default settings. Significant p values were corrected to control for the false discovery rate of multiple testing at 0.05 threshold. All TRAP-seq data were subjected to the work flow described in https://gensat.rockefeller.edu/heintzp30/Bioinformatics_Flow_Chart.jsp

Quantitative RT-PCR

As described previously[10], 10 ng of cDNA was used for each qPCR reaction and all samples were run in triplicate. Q-PCR was carried out using an Applied Biosystems 7900HT system.

Taqman assays were purchased from Applied Biosystems and performed as follows. Tagman assay ID for each gene; p11 (Mm00501457_m1). All data were normalized to TaqMan Rodent GAPDH Control, and relative expression levels between conditions were calculated by the comparative CT (2-*ΔΔ*CT) method.

Magnetic resonance imaging (MRI)

Imaging. Imaging was performed on a 7.0 Tesla 70/30 Bruker Biospec small animal MRI system (Bruker Biospin, Billerica, MA) with 450 mT/m gradient amplitude and a 4500 T/m/s slew rate. The animals were anesthetized with isoflurane in oxygen and fixed in the MRI using a nose cone and bite ring. A volume coil was used for transmission and a quadrature surface coil for reception.

Anatomy. Anatomical images were acquired using a T2 RARE sequence with a RARE factor of 16, 20 averages of 24 axial slices were acquired with a field of view of 20 × 20 mm, spatial resolution of 0.1 × 0.1 × 0.5 mm3, matrix size of 192 × 192, echo time TE of 9.4 ms, effective TE of 56 ms, and recovery time TR of 4.1 s. Fat and motion suppression parameters were used. The covered volume consisted of the entire brain excepted the olfactory bulb. This resulted in a total scan time for the anatomy images of 16 min.

CSF Flow. Using a FLOWMAP phase contrast sequence, eight averages of nine axial slices, centered at the anatomical image volume, were acquired with the same field of view and in-plane resolution as the anatomical images, with slice thickness of 1.0 mm, TE = 5.64 ms, TR = 15.1 ms, and flip angle = 20°. Velocity mapping with flow encoding in slice direction was used with a velocity encoding coefficient of 4 cm/s, directed away from the cerebellum. The total scan time was 7 min.

Analysis. The CSF flow images were analyzed with ParaVision 5.1. software (Bruker Biospin). Analysis on slices of the cerebral aqueduct from cerebellum forwards where aqueduct looked more or less round and not yet inverted y shaped. Freehand ROIs on slightly smoothed images, tracing the darkened area, meaning negative flow, towards the cerebellum. The anatomical images of the all ventricles were quantified with Image J software (NIH).

Ultrastructural analysis

Electron microscopy and transmission electron microscopy. Mice were perfused in a fixative containing 2% paraformaldehyde and 2.5% glutaraldehyde (GA) in 0.075M sodium cacodylate buffer pH 7.4 and post-fixed overnight with the same fixative at 4°C. The mouse brain tissue, which is included the lateral ventricle was placed in a mold and sliced into 1 mm thickness. The selected slices with structure of interest were re-fixed with 2.5% GA and with 0.1% tannic acid for 1 hour and with 2.5% GA in the buffer overnight. The slices were post-fixed with 1% osmium tetra-oxide, 0.4% potassium ferrocyanide for 1 hour, followed by *en bloc* staining with 1% uranyl acetate overnight. Sections were subsequently dehydrated with a graded ethanol series, infiltrated with Eponate12 resin (Ted Pella) and embedded with the resin. Thin sections (70-nm thick) of the ventricular–subventricular zone (V–SVZ) of lateral ventricle were cut by an ultramicrotome (Ultracut E, Leica) and analyzed by JOEL 100CX TEM with a tungsten filament at 80 kV with a digital imaging system (XR41-C, Advantage Microscopy Technology).

Immuno-electron microscopy. Mice were perfused with a fixative, containing 4% paraformaldehyde in 0.075 M sodium cacodylate buffer pH 7.4, post-fixed overnight and then cut into 50-µm-thick sections using a vibrating microtome (Vibratome VT100, Leica). The lateral ventricle sections were processed for immunostaining with ABC method (VECTASTAIN® Elite® ABC-HRP Kit) by following the vendor’s instruction and silver enhancement as described previously[47]. Subsequently, sections, which were included the V–SVZ of lateral ventricle, were processed for EM and analyzed by TEM as described above.

Scanning electron microscopy. Mice brains were fixed in a fixative, containing 2% paraformaldehyde and 2.5% glutaraldehyde in 0.075 M sodium cacodylate buffer pH 7.4. Tissue were dehydrated by a graded series of ethanol (50%, 75%, 95%, and 100% 3 times) and followed by a critical point dry (CPD), which was initiated in the ethanol-filled chamber and replaced with carbon dioxide liquid in the CPD chamber (Autosamdri A-815, Tousimis). 2 nm thickness conductive coating was applied over the dried samples with iridium to ~2 nm (ACE600, Leica). The samples, which were included the V–SVZ of lateral ventricle, were examined under a SEM

(LEO 1550; Carl Zeiss) with a field-emission electron gun and operation/data acquisition software (Smart SEM version 5).

Analysis. TEM and SEM images were analyzed with Image J software (NIH) for the cilia morphology, such as cilia diameter (TEM), cilia number, length and direction (SEM) on the V-SVZ of LV ependymal cells and averaged on each group. To analysis cilia direction, eight radial lines, 45 degrees of each were draw from the middle part of cilia on each cell on SEM images. The relative distribution of cilia within 45 degrees or the other 315 degrees were counted as the unidirectional or multidirectional cilia orientation, respectively, and were calculated as the percentage of cilia direction per cells and averaged on each group. All EM studies were conducted at The Rockefeller University Electron Microscopy Resource Center.

Immunohistochemistry

Mouse brains were perfused transcardially with cold PBS, followed by 4% paraformaldehyde (PFA) and post-fixed in the same solution overnight at 4°C, as described previously[10, 28]. The mouse brains were coronally cut into 40-µm-thick sections with a vibratome (VT 1000S, Leica). Free-floating sections were washed three times with 0.1 M PBS containing 0.1% Triton X-100 in PBS-T, pH 7.4, for 15 min each time and permeabilized with PBS-T in 2% normal goat serum, 2% normal horse serum, and 2% BSA for 1 h. Frozen human postmortem brain, 14-µm-thick, sections from major depressive disorder (MDD) patients and non-affected controls (CON), were provided by Stanley Medical Research Institute (SMRI). See **Supplementary Table S1** for the detailed sample information. Upon arrival, tissue was stored at -80°C freezer until use. Human brain sections were thawed for 20 min on foil. Sections were fixed in 4% PFA at 4°C, washed three times for 5 min in PBS, and blocked in PBS + 10% heat-inactivated normal donkey serum for 30 min at room temperature. After blocking, sections were incubated with the primary antibodies diluted in the blocking buffer. Immunohistochemistry was done using the following antibodies: anti-mouse p11 (goat polyclonal, 1:200, R&D systems), anti-eGFP (chicken polyclonal, 1:500, Abcam), anti-human p11 (goat polyclonal, 1:200, R&D systems), anti-FoxJ1 (mouse monoclonal, 1:500, eBioscience), anti-S100β (rabbit polyclonal 1:400, Dako) and anti-acetylated tubulin (mouse monoclonal, 1:1000, Sigma Aldrich). After 24 hours incubation at 4°C, sections were washed, and incubated with Alexa-fluor-conjugated secondary antibodies

(1:500, Invitrogen). Slices were washed three more times in PBS-T for 15 min each, incubated in Autofluorescence Eliminator reagent (Millipore) according to the manufacturer's instructions for human slides and mounted with Vectashield mounting medium with DAPI (Vector Laboratories) onto microscope slides.

Fluorescence in situ hybridization (FISH)

FISH (RNAscope®) was performed using the RNAscope® Multiplex Fluorescent Assay (Advanced Cell Diagnostics). Frozen cryostat sections (12-µm-thick, Leica) from perfused (4% PFA in PBS) mouse brain were washed one time with PBS and boiled (~80°C) in 1x Target Retrieval Reagent (Advanced Cell Diagnostics) for 5 min. Afterwards, the slides were washed two times with distilled water and dehydrated with 100% ethanol. Next, protease III Reagent (Advanced Cell Diagnostics) was applied for 30 min at 40°C. Human fresh frozen sections (14-µm-thick), were post-fixed in 4% PFA for 15 min at 4°C, dehydrated in graded alcohols and exposed to Protease IV (Advanced Cell Diagnostics) for 30 min at room temperature. Subsequently, both perfused mouse and fresh frozen human sections were hybridized with p11 probes (mouse, Mm-S100a10, 410901; human, Hs-S100a10, 506421; Advanced Cell Diagnostics) respectively, for 2 h at 40°C. The hybridization step was followed by standardized steps of amplification (Amp 1-FL 30 min at 40°C, Amp 2-FL 15 min at 40°C, Amp 3-FL 30 min at 40°C, Amp 4C-FL 15 min at 40°C). The last amplification step was followed by immunofluorescent staining with primary antibodies and Alexa-fluor-conjugated secondary antibodies. Then the slides were mounted with Dako fluorescent mounting medium (Agilent Technologies). Sections were imaged on a Carl Zeiss LSM 880 confocal microscope (Carl Zeiss AB) using a 63× oil immersion objective. Z-stacks of 7-10 µm thickness were obtained in each caption.

Cell intensity

The intensity of eGFP and p11-immunolabeled ependymal cells was quantified with ImageJ software (NIH). Three to five coronal sections that included lateral ventricles per mouse and human were quantified and averaged for each group. Fluorescence images for lateral ventricles from mice and humans, third ventricle and fourth ventricle from mice were acquired using a Zeiss LSM710 confocal microscope with a ×40/0.50 NA objective (45176.65 um^2; 262144 pixels). Background autofluorescence was accounted for by applying an equal cut-off threshold

to all images. All imaging and analyses were performed blind to the experimental conditions. Data were analyzed by ANOVA test or Student's t-tests and later graphed using Microsoft Excel or GraphPad Prism Software.

Viruses

As described previously[10], for Cre-mediated recombination/inversion of the flanked p11 as DIO (double-floxed inverse ORF) viruses. AAV Vector production of the AAV1 serotype was performed by the University of Pennsylvania vector core. AAV1-EF1a-DIO-eYFP-WPRE-hGH was used as the control vector. AAV1-EF1a-DIO-p11-WPRE-hGH vector construction for the overexpressing of p11. Double floxed AAV constructs were generated by insertion of the inverted p11 expression cassettes between double lox 2722 and lox P incompatible sites (DIO). In the absence of Cre expression, the p11 or eYFP were not produced. In the presence of Cre expression, the transgene was FLEXed, leading to the expression of p11 or eYFP. The titers (genome copies per milliliter) of the AAVs were as follows: $2.16e^{13}$ for AAV1-EF1a-DIO-eYFP-WPRE-hGH (AAV_eYFP) and $3.51e^{12}$ for AAV1-EF1a-DIO-p11-WPRE-hGH (AAV_p11).

Stereotaxic surgery

All stereotaxic injections were carried out on an Angle Two stereotaxic frame for mouse with motorized nanoinjector (Leica). 10-week-old male mice were anesthetized with ketamine and xylazine and stereotaxically injected with AAV1-EF1a-DIO-eYFP-WPRE-hGH and AAV1-EF1a-DIO-p11-WPRE-hGH intracerebroventricularly (I.C.V., AP: 0.02 mm; ML: ± 0.89 mm; DV: -2.53 mm from bregma). The total injection volume was 1 µl. All injections were performed at a rate of 0.15 µl/min using Hamilton syringes (33 gauge) and the needle was kept in place for an additional 5 min. After 14 days of injection, depression-like behavioral tests and MRI were performed.

Behavioral assessments

All the behavioral tests were performed during the light cycle in a dedicated sound-proof behavioral facility by experimenters blind to treatment and genotype information, as described previously[10, 11]. Mice were brought to the testing room 30 min before the start of each behavioral

test and remained in the same room through the test. At all times, sound was masked with 60–65 dB white noise.

Tail suspension test. Mice were suspended individually by their tails. The rod was fixed 50 cm above the surface of a table covered with a safety mat in a sound-isolated room. The tip of the tail was fixed using adhesive Scotch tape; the duration of the test was 5 min. The test session was videotaped and immobility scored by using automated TST/FST analysis software from Clever Systems.

Forced swim test. Mice were placed in a glass cylinder (height: 30 cm, diameter: 16 cm) containing water at 24°C and a depth of 14 cm so that they could neither escape nor touch the bottom. Mice were forced to swim for 6 min. The animals were habituated for the first 1 min and behavior was monitored over the next 5 min. A 6 min test session was videotaped and immobility scored by using automated TST/FST analysis software from Clever Systems.

Novelty-suppressed feeding test. After 24 hours food-deprivation (water was provided ad libitum), mice were tested in the NSF test, At the end of this time, a single 2 x 2 cm oval food pellet was placed on a circular piece of white filter paper (150 mm diameter) positioned in the center of the open field (40 x 40 x 40 cm). Each mouse was placed in a corner of the open field. The latency to first bite the lab chow pellet and consumption over 15 min was recorded. Immediately after the mouse began to eat the chow, the tested animal was placed in its home cage alone with a weighed piece of chow for 30 min. At the end of this period, the amount of food consumed was determined by weighing the piece of chow.

Locomotion test. Locomotor activity was measured in the open field of a Plexiglas chamber (40 x 40 x 40 cm). Each mouse was placed in the corner of the open field, and locomotion was recorded for the indicated period of 30 min. An automated Superflex software (Accuscan Instruments) was used to measure the total distance traveled across a session. The measures were automatized using two rows of infrared photocells placed 20 and 50 mm above the floor, spaced 31 mm apart. Photocell beam interruptions were recorded on a computer using the Superflex software.

Bioinformatics

RNAseq analysis and geneset enrichment. Ependymal RNA-seq reads were aligned with STAR software[48], and differential expression analysis was performed by EdgeR[49]. Gene set enrichment analysis was performed by PAGE (Parametric Analysis of Gene Set Enrichment)[50] and conducted on mouse Gene Ontology Biological Processes and expert-curated terms (only terms with 10-500 genes were included in the analysis). Annotated PCP genes were referred to a previous report[33].

Construction of lateral ventricular ependymal cell-specific functional network. Following the scheme of a previous study[51], we constructed a human lateral ventricular ependymal cell network to represent the functional interaction pattern of each pair of genes in lateral ventricular ependymal cells. The network was built by a regularized Bayesian classifier which integrates thousands of large scale datasets (e.g. co-expression, protein-protein interaction, etc. additional datasets[38] are listed in **Supplementary Table S6**). Positive gold standards for network integration were gene pairs both of which are expressed in lateral ventricular ependymal cells and have known functional interactions, while negatives were pairs either not co-expressed in ependymal cells or not functionally connected[51, 52]. Specifically, to represent lateral ventricular ependymal cell specific functions, lateral ventricular ependymal specific genes were determined by the union of genes highly expressed in lateral ventricular ependymal cells in data presented in this study, ependymal cell markers identified in previous study[52], as well as expert curated markers; gene markers from the other major brain cell types (neuron, microglia, astrocyte, oligodendrocyte) were filtered out to ensure ependymal cell specificity. The network was evaluated by cross validation, as well as the ability to use network neighborhood to recapitulate the differential expression pattern of p11 knockout in this study. Mouse genes were mapped to human based on the combination of sequence and functional conservation using the Functional Knowledge Transfer (FKT) approach[53].

Clustering the depression-associated ependymal cell network. In order to identify depression-associated functional modules of the ependymal cells, we created a subset of the ependymal cell-specific functional network containing the top 1,000 depression-associated genes from our genome-wide ranking and all the edges between them. Then, we used an approach based on

shared k-nearest-neighbors (SKNN) and the Louvain community-finding algorithm to cluster the network into distinct modules of tightly connected genes. The SKNN-based strategy has the advantages of alleviating the effect of high-degree genes and accentuating local network structure by connecting genes that are likely to be functionally clustered together. Cytoscape used to layout the network in **Supplementary Fig. S13**. The entire table of enriched GO terms is provided in listed in **Supplementary Table S7**.

SUPPLEMENTARY FIGURE LEGENDS

Supplementary Figure S1. p11 expression in ependymal cells in the brain.
a, Immunofluorescence image illustrating p11-positive cells ($EGFP^+$) in the lateral ventricle (LV), dorsal and ventral third ventricle (d3V, v3V) and choroid plexus (ChP). Scale bars, 200 μm for LV, ChP and d3V; 500 μm for v3V. **b**, Co-localization of ependymal p11 ($EGFP^+$) with S100β (blue), a marker of ependymal cells, in the fourth ventricle (4V) and cerebral aqueduct (Aq) from p11-EGFP mice. Scale bar, 20 μm.

Supplementary Figure S2. p11 mRNA expression in ependymal cells in the brain.
a-**f**, Fluorescence in situ hybridization using mouse p11 probe (p11-FISH) images displays co-localization of ependymal p11 (yellow) with FoxJ1 (red) and S100β (blue), ependymal cell markers, in the lateral ventricle (LV, **a**), dorsal third ventricle (d3V, **b**), ventral third ventricle (v3V, **c**), forth ventricle (4V, **d**), cerebral aqueduct (Aq, **e**) and choroid plexus (ChP, **f**). Scale bar, 30 μm.

Supplementary Figure S3. p11 expression in ependymal cell-specific bacTRAP mouse.
a, Immunofluorescence image illustrating $EGFP^+$ signals from ependymal cell-specific bacTRAP (Dcdc2a-L10a/EGFP) mouse line. Scale bar, 1 mm. **b**, Co-localization of EGFP and p11 (red) in ependymal cells in the ventricular-subventricular zone (V-SVZ) of lateral ventricle from ependymal cell bacTRAP mouse line. Scale bar, 5 μm.

Supplementary Figure S4. p11 in ependymal cells is regulated by chronic restraint stress and antidepressant. Immunofluorescence image and quantification display p11 expression in LV ependymal cells from non-stressed control (CON) and chronic restraint stressed (RST) mice with or without imipramine (Imi). Scale bar, 10 μm (n = 3, CON; n = 4, RST; and n = 6, RST+Imi). # $P < 0.05$, compared to CON; * $P < 0.05$, compared to RST, ANOVA test. Data are means ± s.e.m.

Supplementary Figure S5. Chronic isolation stress induces loss of p11 in ependymal cells and depression-like behaviors. a, Immunofluorescence image and quantification display p11-positive ($EGFP^+$) ependymal cells from control (CON) and chronic isolation stressed (IS) mice

(n = 5 for each group of CON and IS). Scale bar, 5 μm. **b** and **c**, Depression-like behaviors from control and isolation stressed mice, as measured by immobility time in tail suspension test (TST, **b**) and forced swim test (FST, **c**), (n = 8, CON; n = 16, IS). *P < 0.05, Student's t-test. Data are means ± s.e.m.

Supplementary Figure S6. Translational profiling for p11-associated genes in ependymal cells. Heat map displaying ependymal cell translational profiling from WT (Dcdcd2a-L10a/EGFP) and p11 KO (p11 KO x Dcdc2a-L10a/EGFP) bacTRAP mice (n = 12, WT; n = 8, p11 KO).

Supplementary Figure S7. p11-mediated signaling pathway in ependymal cells. Rank based gene set enrichment displaying p11-regulated LV ependymal cell gene differential expression, including planal cell polarity (PCP, red box), presented as z-score (positive z-scores indicate up-regulation of processes in WT compared to p11 KO mice).

Supplementary Figure S8. p11 KO mice exhibits cilia disorientation. a-d, Scanning electron microscopy (SEM) images display ependymal cilia orientation (**a**), quantification of unidirectional cilia orientation (**b**), cilia number (**c**), and cilia length (**d**) in the ventricular–subventricular zone (V–SVZ) of lateral ventricle from p11 KO mice and WT littermates (n = 20 cells for **b**; n = 34 cells for **c**; n = 43 cilia for WT and n = 64 cilia for p11 KO for **d** from 3 mice in each group). Scale bar, 5 μm. ***P < 0.001, Student's t-test. Data are means ± s.e.m.

Supplementary Figure S9. Reduced p11 expression in ependymal cells from stressed mice exhibits cilia disorientation 3. a-d, Scanning electron microscopy (SEM) images display cilia orientation (**a**), quantification of multidirectional (**b**), unidirectional cilia orientation (**c**), and cilia number (**d**) in the V–SVZ of lateral ventricle from non-stressed control (CON) and chronic restraint stressed (RST) mice (n = 11 cells from 3 mice in each group). Scale bar, 5 μm. **P < 0.01 and ***P < 0.001, Student's t-test. Data are means ± s.e.m.

Supplementary Figure S10. p11 in cilia of ependymal cells from mice and humans. a, Co-localization of p11 ($EGFP^{+}$) and acetylated tubulin (Ac-tub, a marker gene of cilia, red) in cilia

of ependymal cells in the V–SVZ of lateral ventricle from p11-EGFP mice. Scale bar, 5 μm. **b**, Immuno-electron microscopy staining with p11 antibody (p11 IEM) illustrating p11 expression (dot) in cilia. Scale bar, 200 nm. **c**, Co-localization of p11 (green) with Ac-tub (red) in cilia, and merged with DAPI (nuclear DNA, blue) from human ependymal cells in the V–SVZ. Scale bar, 5 μm.

Supplementary Figure S11. Cilia morphology is not different in p11 KO mice. Transmission electron microscopy (TEM) images display cilia axoneme structure, and quantification of cilia diameter from WT and p11 KO mice (n = 11 cilia for WT and n = 15 cilia from p11 KO from 3 mice in each group). Scale bar, 100 nm.

Supplementary Figure S12. Ventricle size is not altered in p11 KO mice. Magnetic resonance (MRI) images and quantification represent ventricle size in the lateral ventricle (LV), third ventricle (3V), cerebral aqueduct (Aq) and fourth ventricle (4V) from WT littermates and p11 KO mice (n = 7 for each group). The loss of p11 does not change ventricle size, suggesting that p11 may not be involved in regulation of choroid plexus CSF production, drainage or reabsorption. Scale bar, 850 μm. Data are means ± s.e.m.

Supplementary Figure S13. Depression-associated ependymal cell-specific functional modules. The network of ependymal cell-specific functional interactions among the top 1,000 depression-associated genes were clustered using a shared-nearest-neighbor–based community-finding algorithm to elucidate several modules of genes. Eight of the clusters that contained 10 or more genes, labeled C1 through C8, were tested for functional enrichment using genes annotated to Gene Ontology biological process terms. Representative processes and pathways enriched within each cluster are presented here alongside the cluster label. The enriched functions provide a landscape of cellular functions potentially dysregulated by depression-associated ependymal genes from mice and humans. All processes are rained with FDR<0.05.

Supplementary Figure S14. Conditional knockout of p11 in ependymal cells induces disruption of ependymal cilia orientation and decreased CSF flow, as well as depression-like behaviors. a, Images showing the co-localization of Tppp3-Cre positive ependymal cells

(Tppp3-Cre; Tppp3-Cre x tdTomato; red) with p11 (green). Scale bar, 10 μm. **b**-**e**, Quantification of CSF flow by MRI (**b**), unidirectional and multidirectional cilia orientation (**c** and **d**) and number of cilia (**e**) from wild-type (WT; $p11^{f/f}$) and ependymal cell conditional p11 knockout (p11 cKO; Tppp3-Cre x $p11^{f/f}$) mice (n = 5 for **b**; n = 11 cells from 3 mice in each group for **c**-**e**). **f**-**i**, Depression-like and anxiety-like behaviors from WT and p11 cKO mice, as measured by immobility time in tail suspension test (TST, **f**), forced swim test (FST, **g**), the latency to feed in novelty suppressed feeding test (NSF, **h**) and the total travel distance in locomotor activity (i) (n = 19, WT; n = 20, p11 cKO). * P < 0.05, **P < 0.01 and ***P < 0.001, Student's t-test. Data are means ± s.e.m.

Supplementary Figure S15. Viral expression of p11 in ependymal cells from p11 cKO mice alleviates cilia disorientation. a, Immunofluorescence images of eYFP (green) in Tppp3-Cre (Tppp3-Cre; Tppp3-Cre x tdTomato; red) mice with the injection of AAV_eYFP (eYFP) into ependymal cells. Scale bar, 10 μm. **b**-**d**, SEM image in the ventricular–subventricular zone (V–SVZ) of lateral ventricle (**b**), quantification of unidirectional cilia orientation (**c**) and number of cilia (**d**) from p11 cKO mice with the viral overexpression of AAV_p11 or AAV_eYFP into ependymal cells. Scale bar, 5 μm, (n = 11 cells from 3 mice in each group). ***P < 0.001, Student's t-test. Data are means ± s.e.m.

Supplementary Figure S16. p11 regulates ependymal cells-CSF flow and stress-induced depression. A graphical abstract simply summarizing key results of the study, 1) High concentration of p11 in ependymal cells is decreased in depression; 2) Loss of ependymal p11 by stress and genetically manipulation induces disoriented PCP, decreased CSF flow and depression-like behaviors; 3) p11 controls PCP genes; and 4) p11 overexpression in ependymal cells ameliorates the pathophysiological and behavioral deficits in depressed mice. CSF, cerebrospinal fluid; EP, ependymal cells; BP, brain parenchyma; PCP, planar cell polarity.

Supplementary Figure S1.

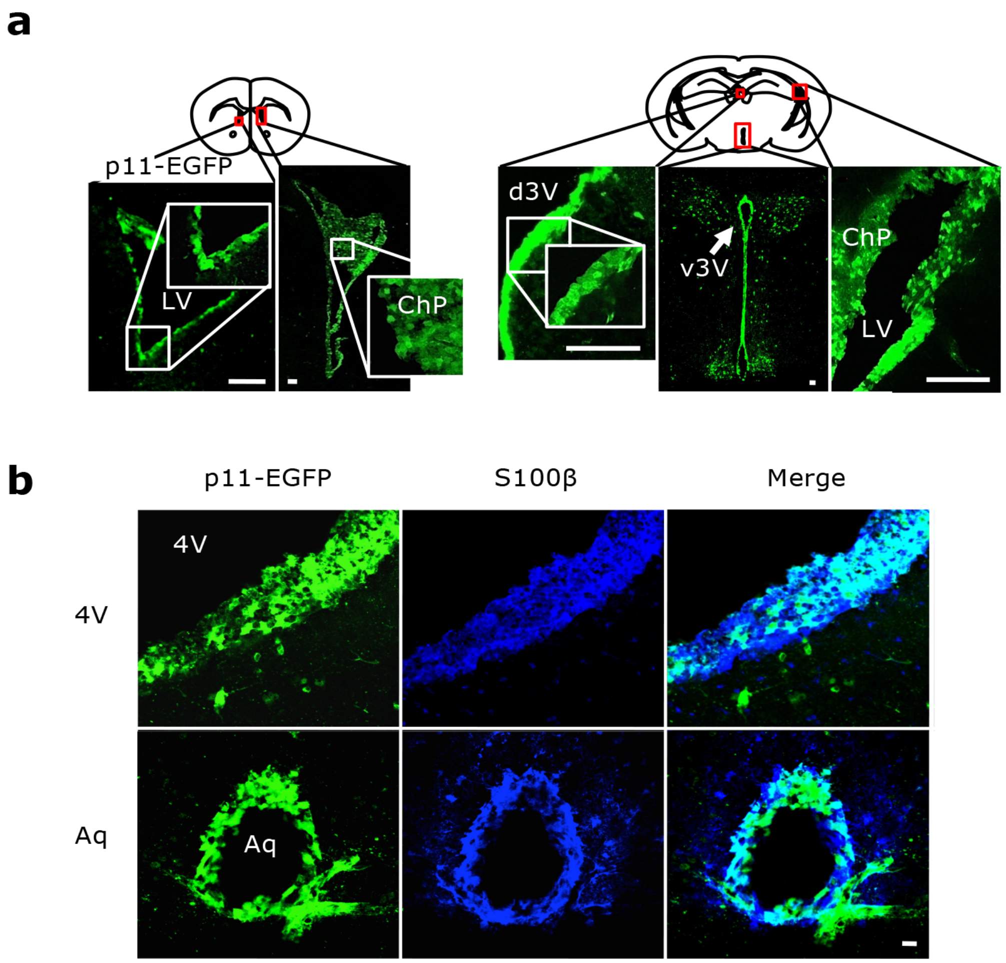

Supplementary Figure S2.

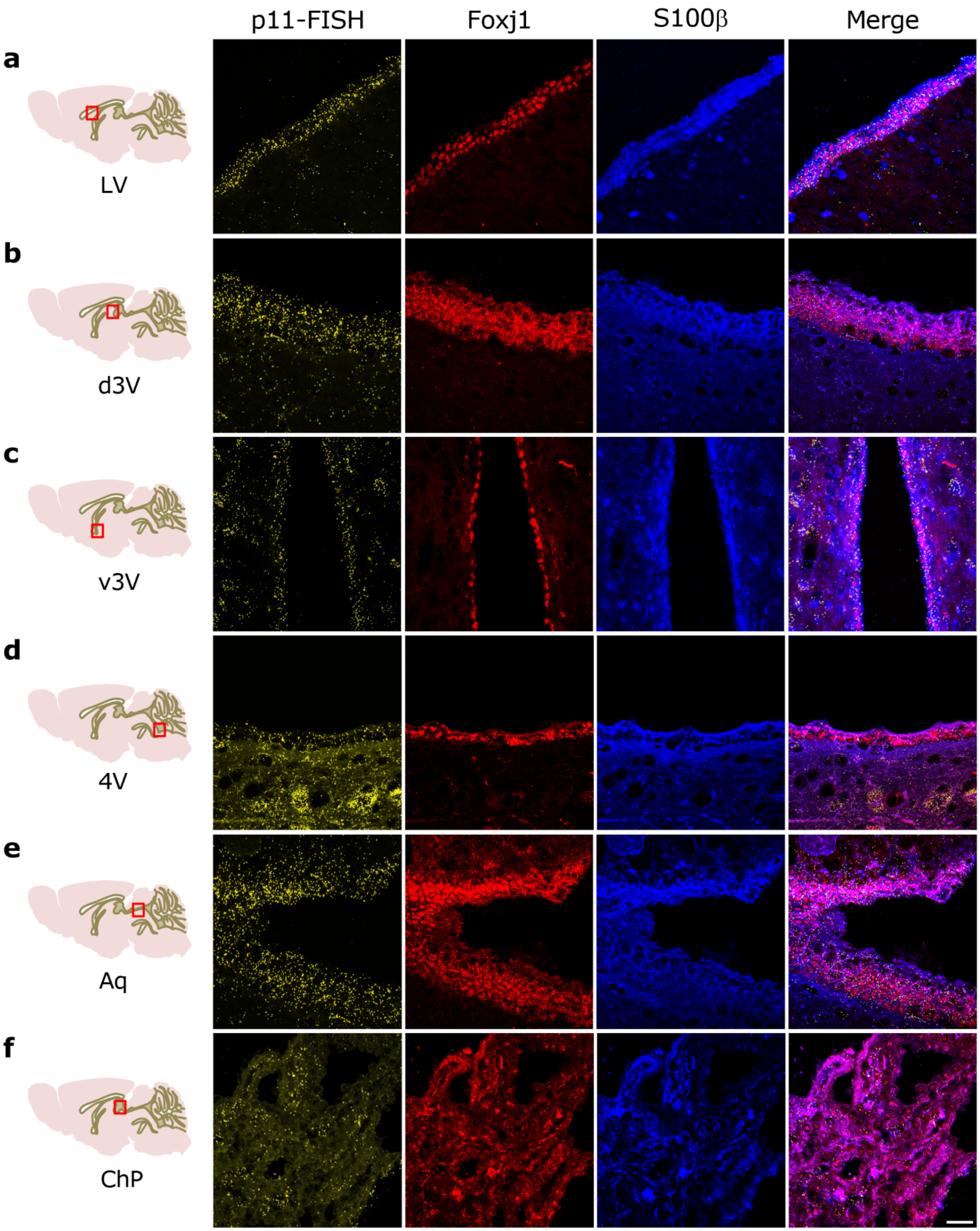

Supplementary Figure S3.

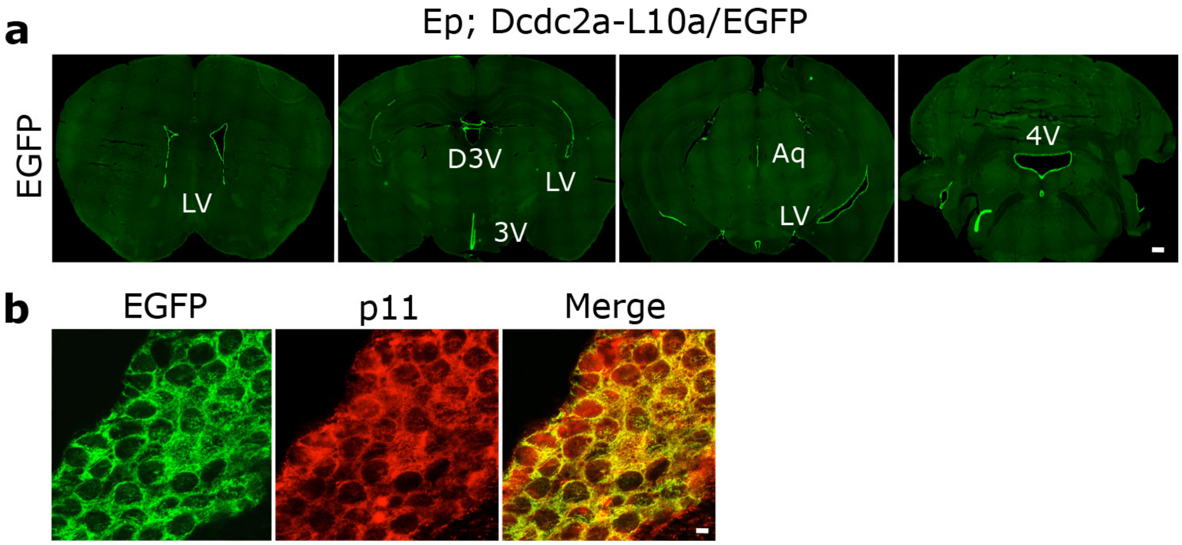

Supplementary Figure S4.

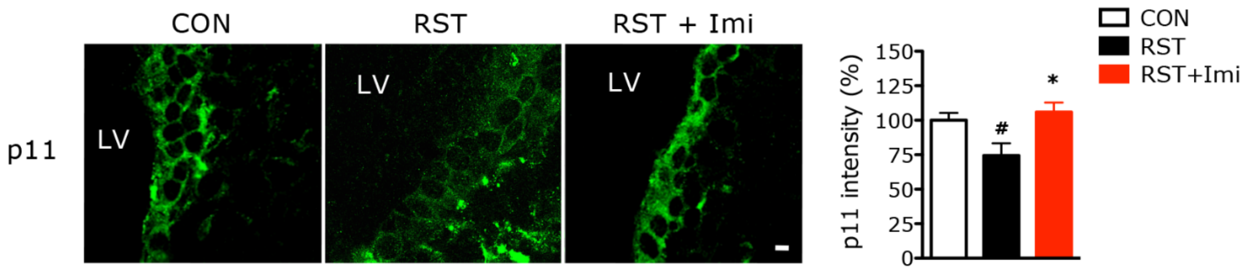

Supplementary Figure S5.

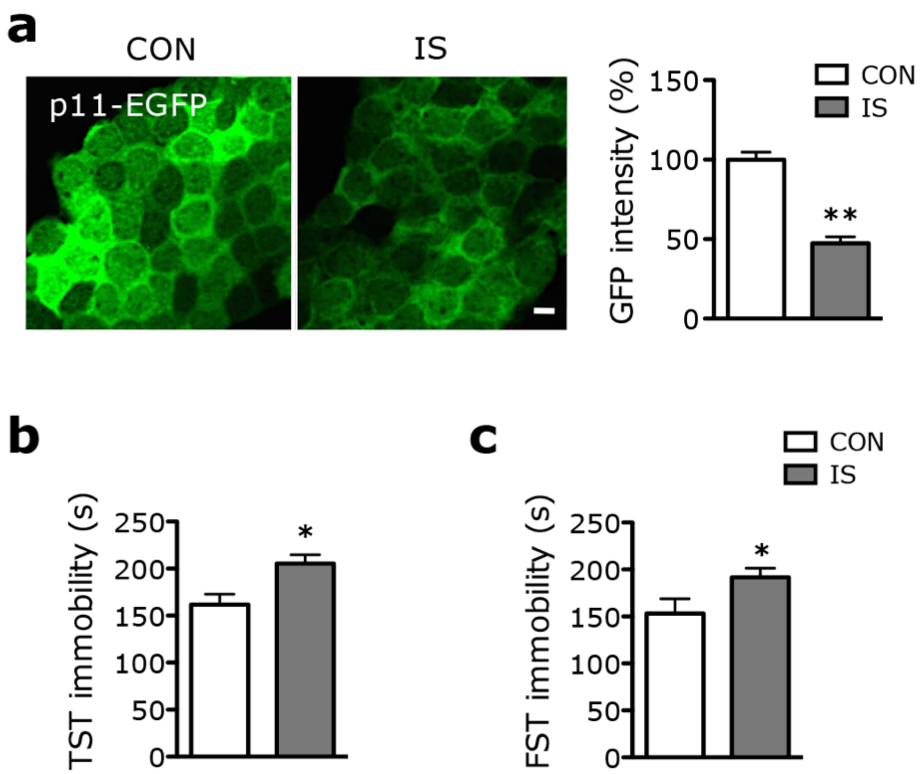

Supplementary Figure S6.

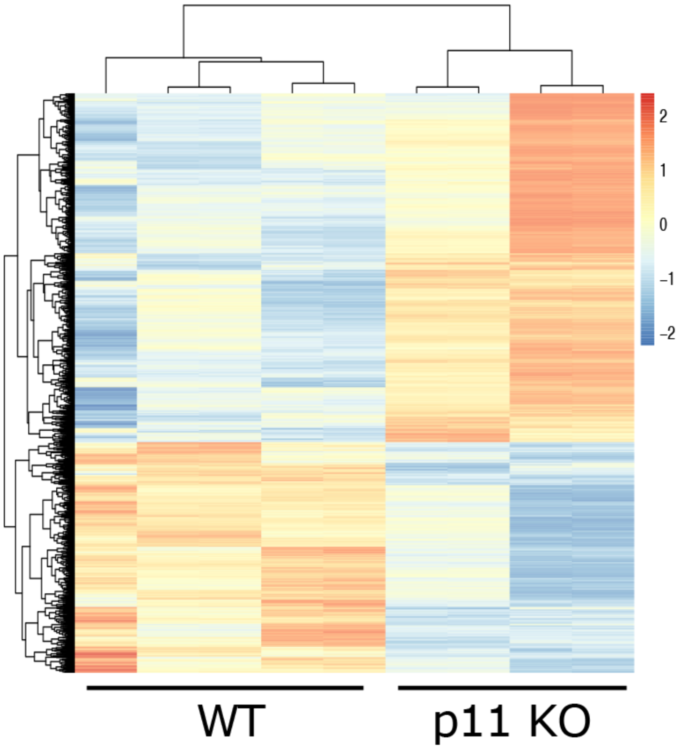

Supplementary Figure S7.

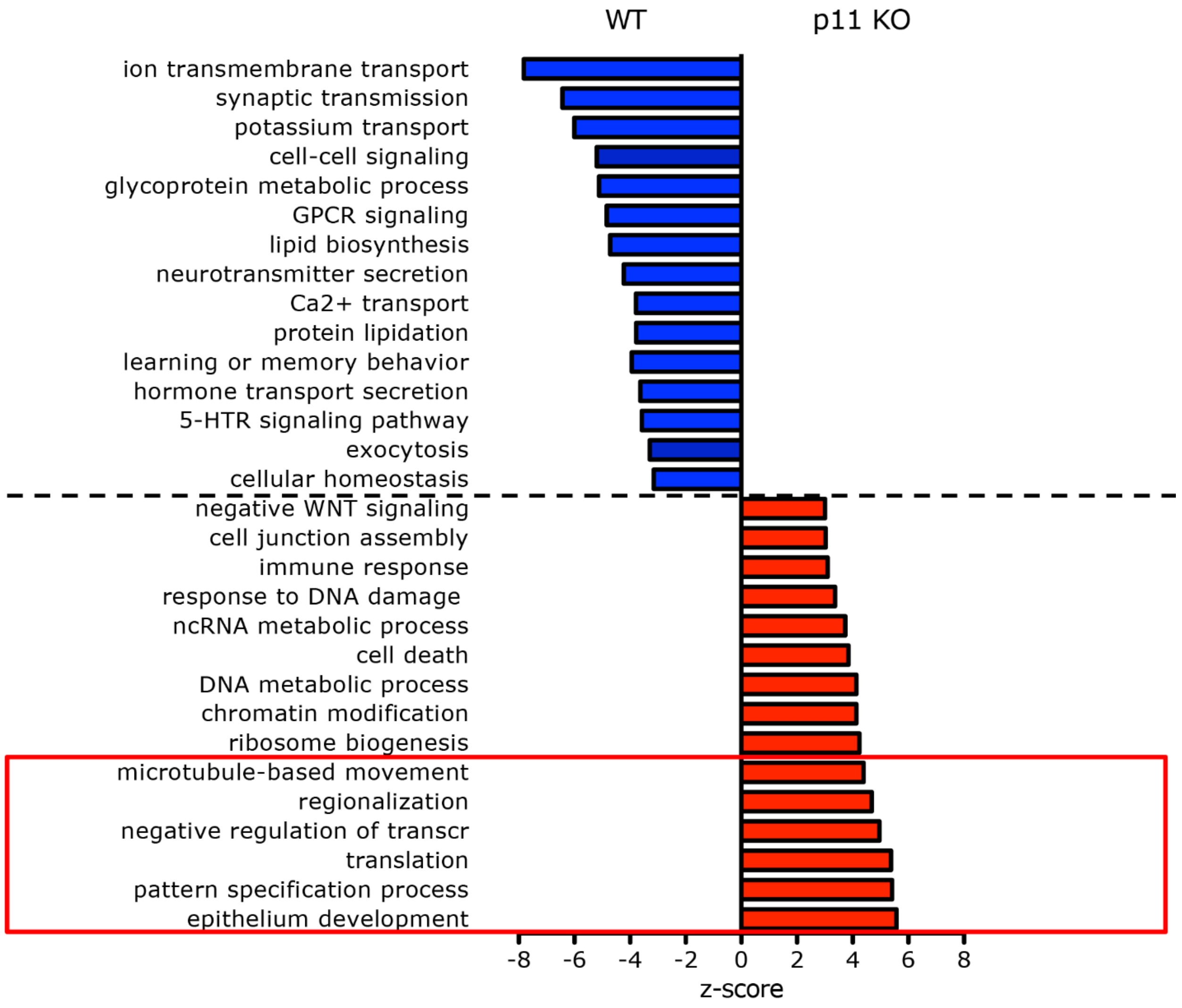

Supplementary Figure S8.

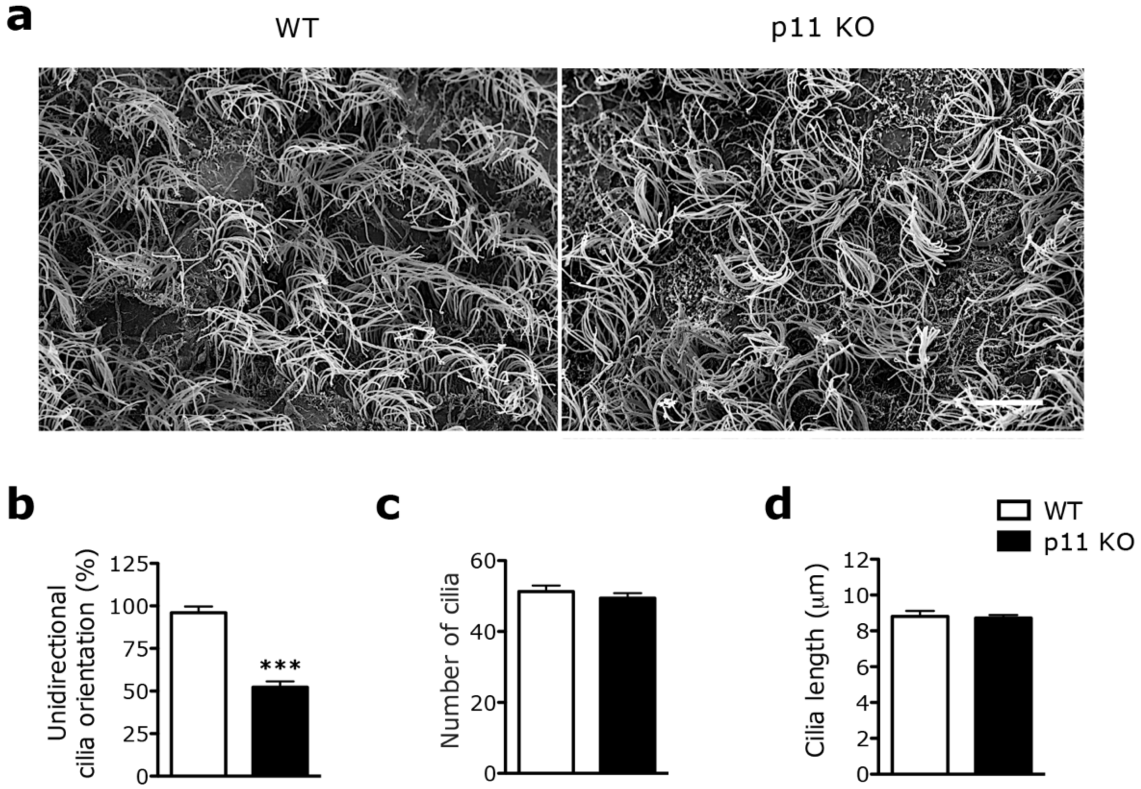

Supplementary Figure S9.

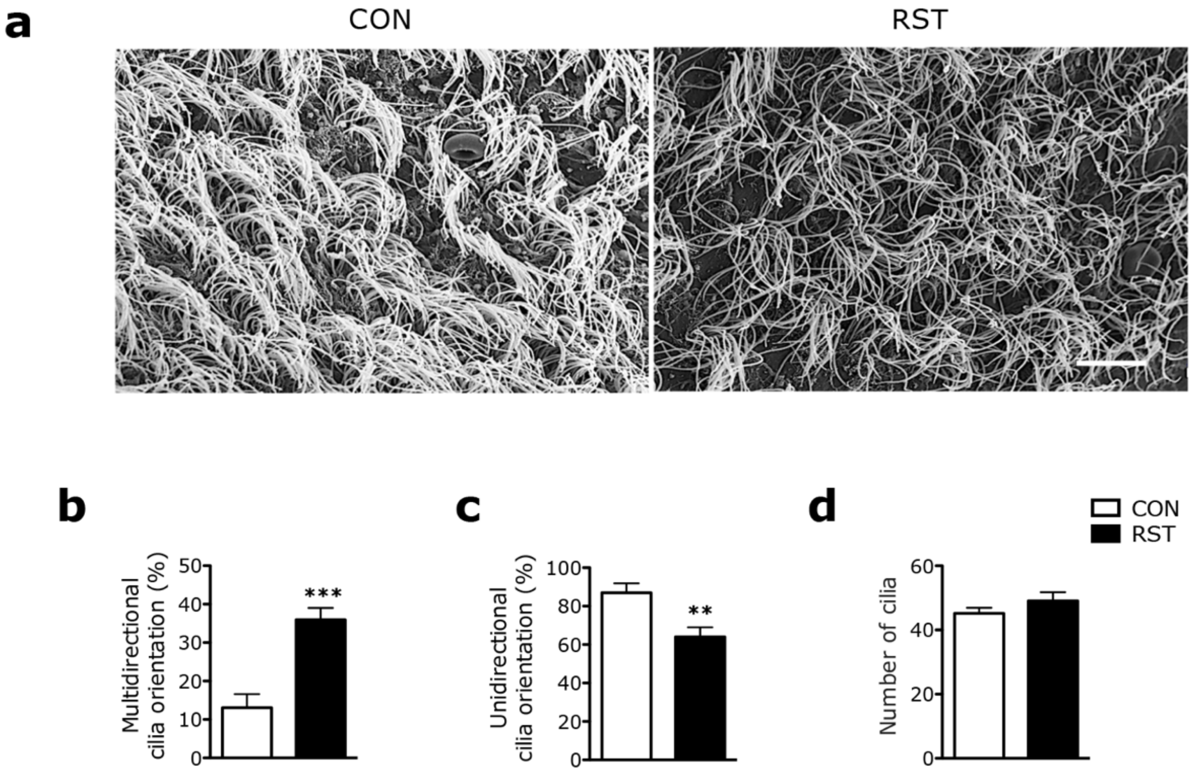

Supplementary Figure S10.

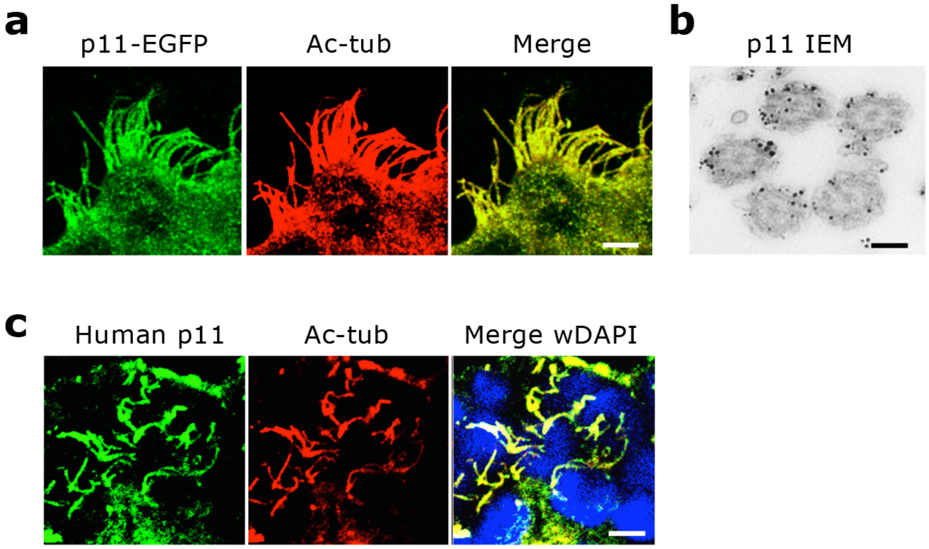

Supplementary Figure S11.

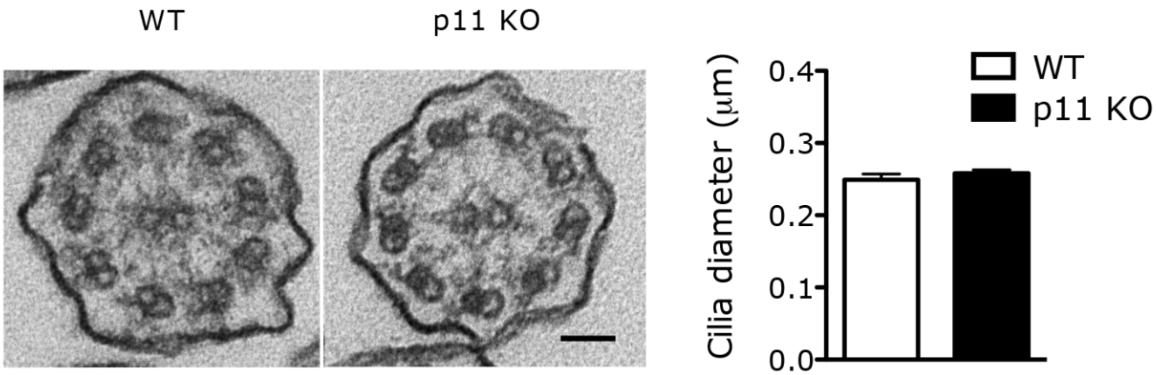

Supplementary Figure S12.

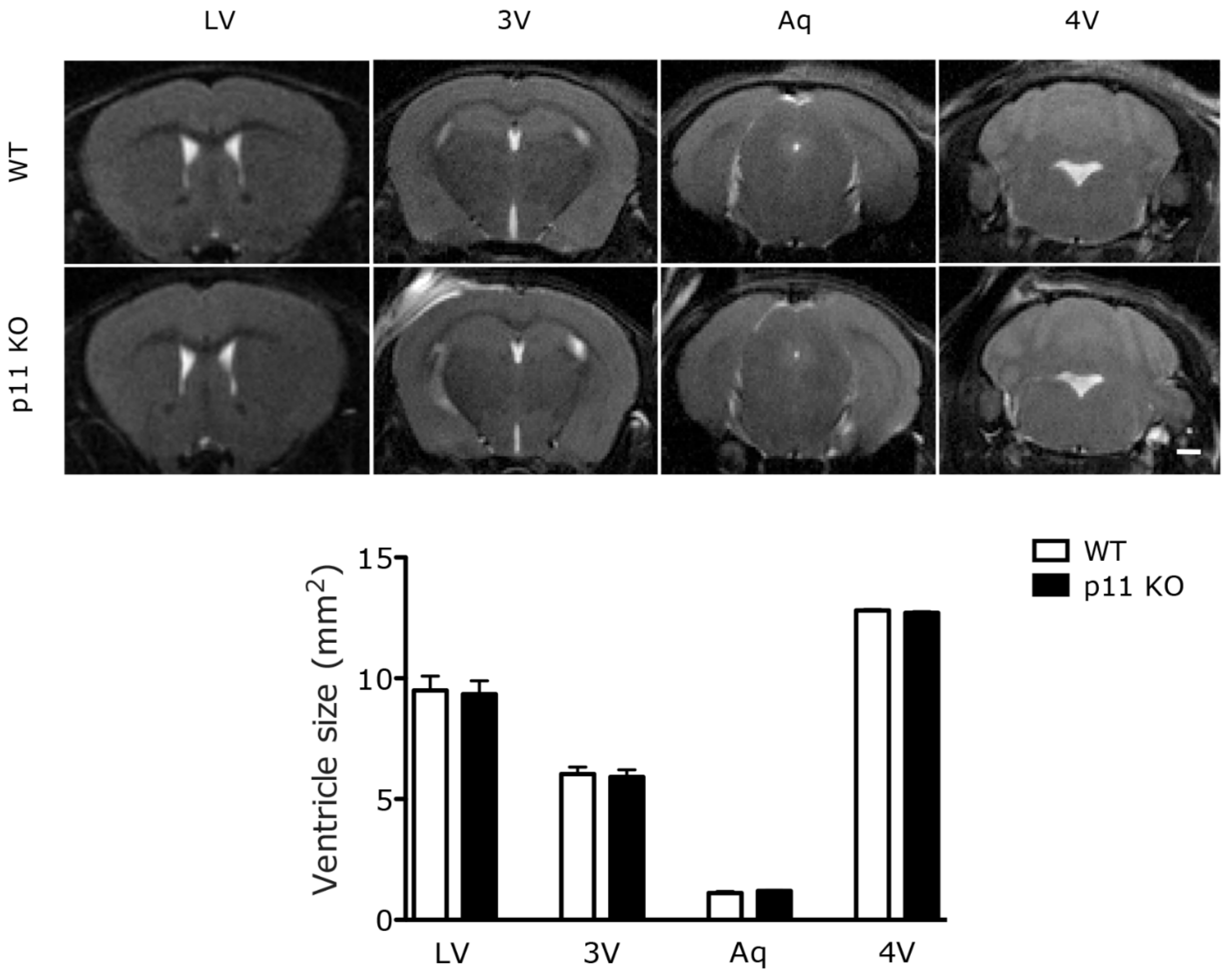

Supplementary Figure S13.

C1
Ca^{2+} and G-protein-cAMP signaling
ATPase activity
Synaptic transmission, neurotransmitters transport
Peptide and hormone secretion
Cognition, learning and memory
Feeding and mating behavior

C2
Embryonic development and morphogenesis
Fluid regulation, respiratory system physiology
Cell migration, neuronal fate regulation
VEGF signaling, intracellular protein transport

C3
Metabolic activity
Myofibril assembly

C4
Hormone secretion
Cell differentiation

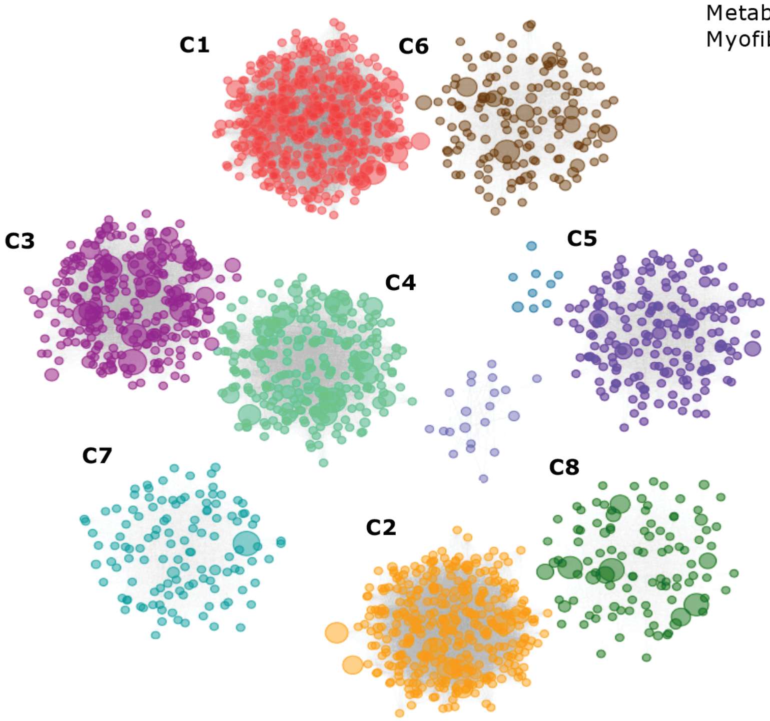

C5
Epithelial cell proliferation
Phospholipase C signaling
TGFβ signaling
Aging

C6
Ca^{2+}, metal and ion transport
Transmembrane transport

C7
Respiratory system process
Hypothalamus development

C8
Toll-like receptors signaling
Stress-activated MAPK signaling
Immune response
Cell adhesion

Supplementary Figure S14.

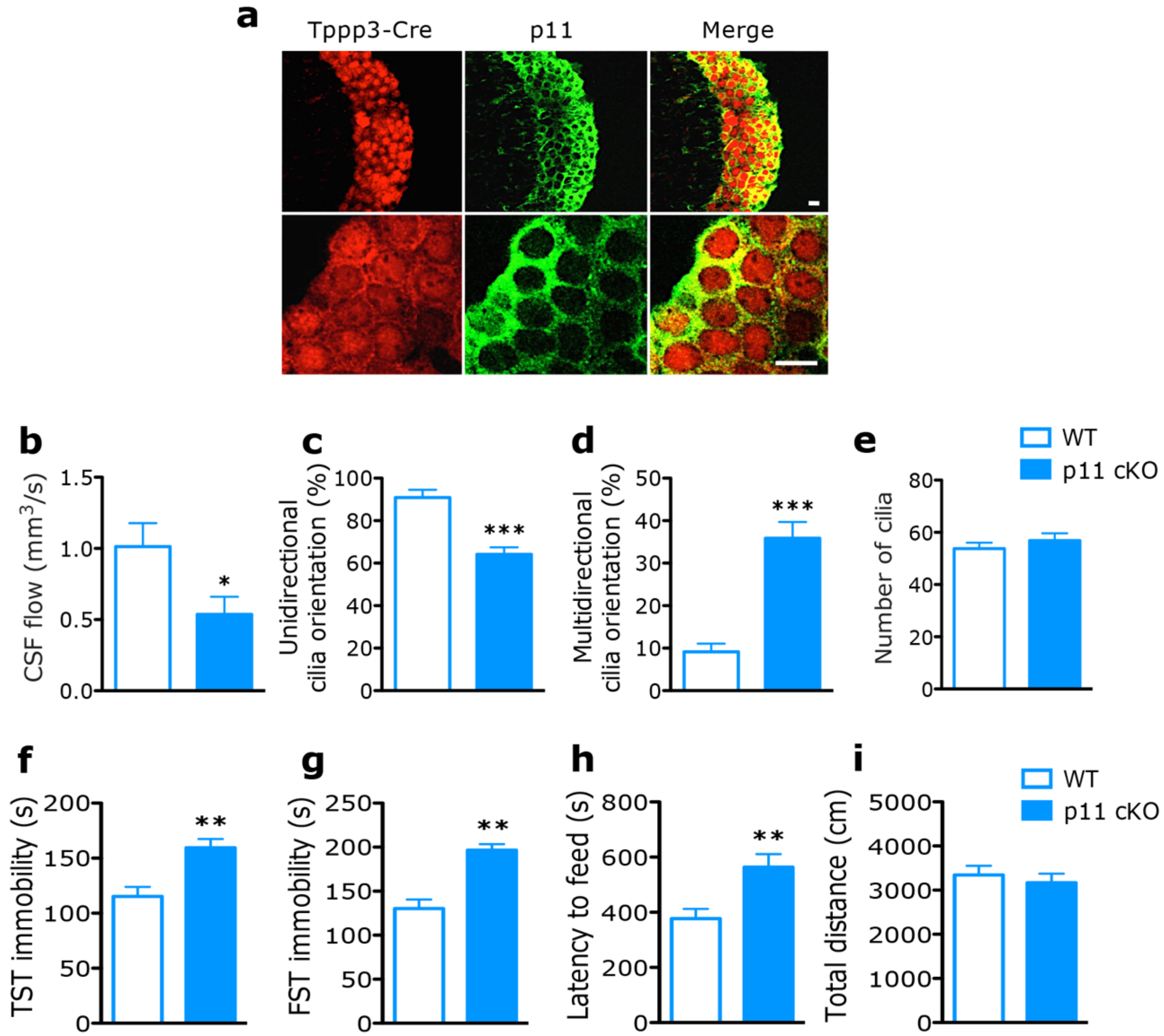

Supplementary Figure S15.

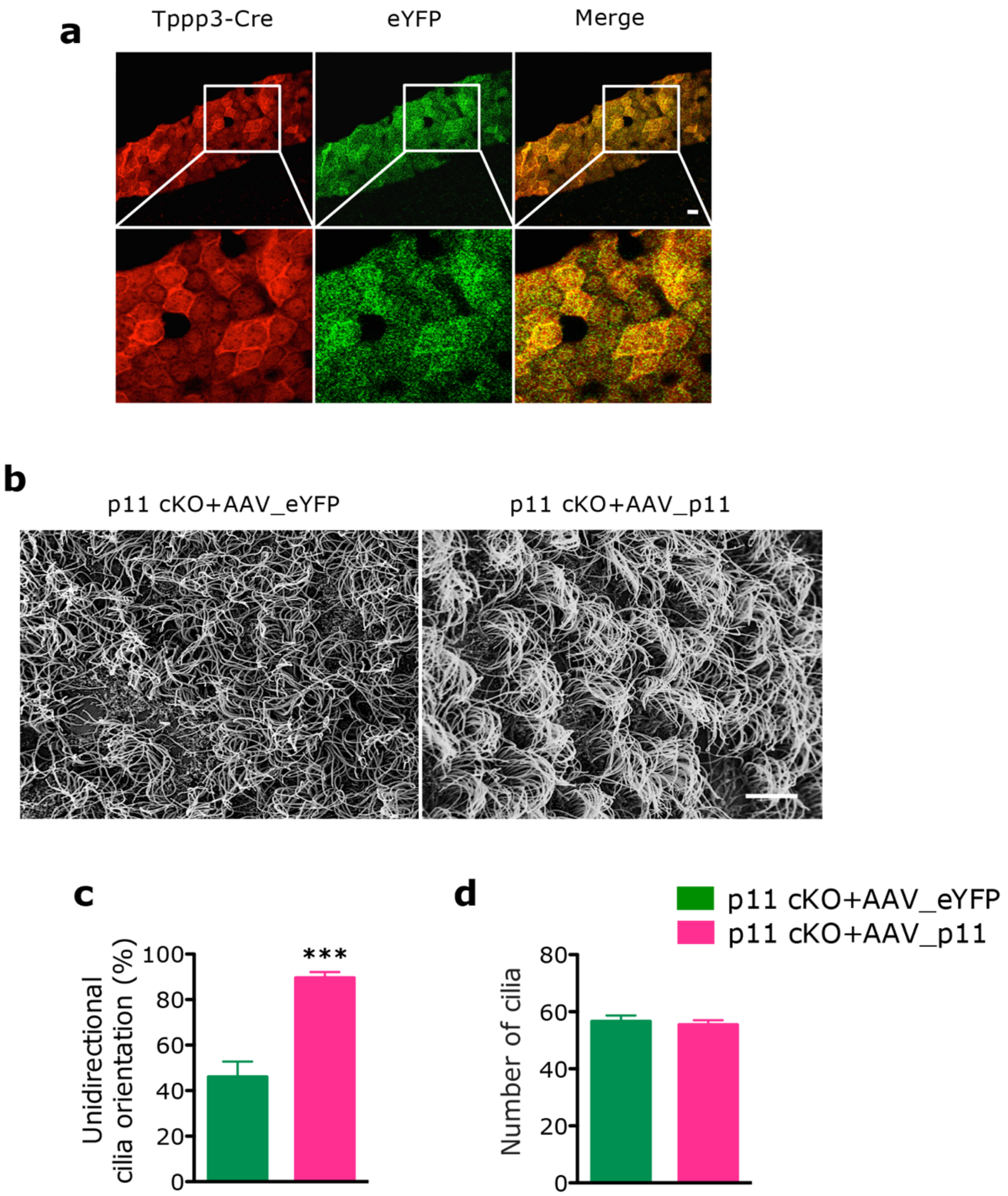

Supplementary Figure S16.

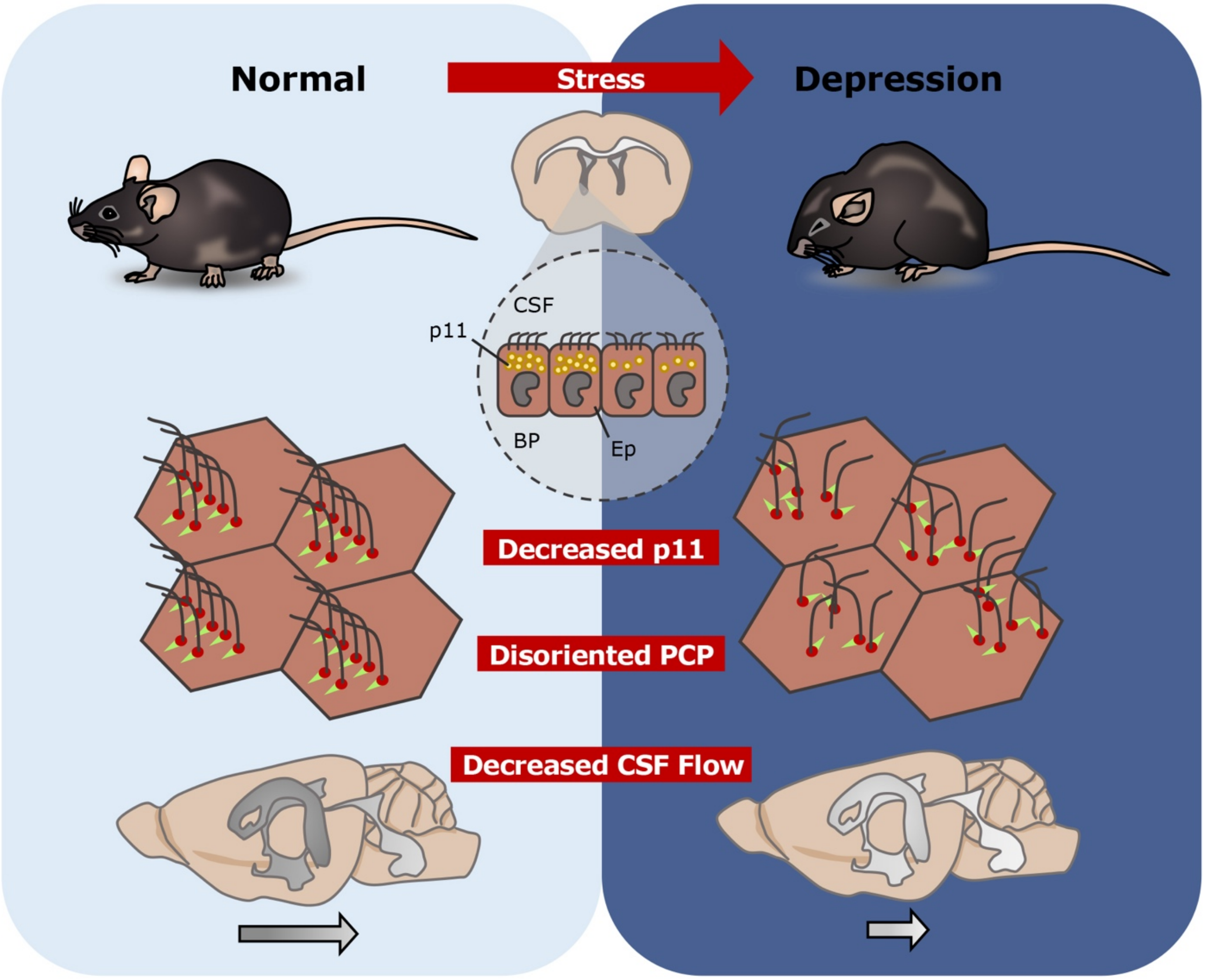

SUPPLEMENTARY REFERENCES

47. Teclemariam-Mesbah R, Wortel J, Romijn HJ, Buijs RM. A simple silver-gold intensification procedure for double DAB labeling studies in electron microscopy. *J Histochem Cytochem* 1997; **45**(4)**:** 619-621.

48. Dobin A, Davis CA, Schlesinger F, Drenkow J, Zaleski C, Jha S *et al.* STAR: ultrafast universal RNA-seq aligner. *Bioinformatics* 2013; **29**(1)**:** 15-21.

49. Robinson MD, McCarthy DJ, Smyth GK. edgeR: a Bioconductor package for differential expression analysis of digital gene expression data. *Bioinformatics* 2010; **26**(1)**:** 139-140.

50. Kim SY, Volsky DJ. PAGE: parametric analysis of gene set enrichment. *BMC Bioinformatics* 2005; **6:** 144.

51. Greene CS, Krishnan A, Wong AK, Ricciotti E, Zelaya RA, Himmelstein DS *et al.* Understanding multicellular function and disease with human tissue-specific networks. *Nat Genet* 2015; **47**(6)**:** 569-576.

52. Campbell JN, Macosko EZ, Fenselau H, Pers TH, Lyubetskaya A, Tenen D *et al.* A molecular census of arcuate hypothalamus and median eminence cell types. *Nat Neurosci* 2017; **20**(3)**:** 484-496.

53. Park CY, Wong AK, Greene CS, Rowland J, Guan Y, Bongo LA *et al.* Functional knowledge transfer for high-accuracy prediction of under-studied biological processes. *PLoS Comput Biol* 2013; **9**(3)**:** e1002957.
